# Trends and Disparities in Mortality from Hereditary Ataxia in United States, 2000–2020: A Retrospective Analysis with Projections to 2050

**DOI:** 10.1007/s12311-026-02046-7

**Published:** 2026-06-29

**Authors:** Muhammad Junaid Iqbal, Fiza Wali, Laraib Israr, Noor Ullah Khan, Hanzala Ahmed Farooqi, Fatima Naveed, Areeba Kabir, Gianluca Morganti, Anastasia Ricci, Michele Menotta

**Affiliations:** 1 Department of Biomolecular Sciences, University of Urbino “Carlo Bo”, Via Saffi 2, Urbino (PU), 61029 Italy; 2 Islamic International Medical College, Riphah International University, Islamabad, 46000 Pakistan; 3 Department of Biological Sciences, International Islamic University Islamabad, Islamabad, 44000 Pakistan; 4 Department of Computer Sciences, University of Porto, Porto, 4099-002 Portugal; 5 Rawal Institute of Health Sciences, Islamabad, 45550 Pakistan; 6 Royal Devon and Exeter Hospital, Royal Devon University Healthcare NHS Foundation Trust, Exeter, UK

**Keywords:** Cerebellar Ataxia, Spinocerebellar Ataxias, Friedreich Ataxia, Ataxia Telangiectasia

## Abstract

Hereditary ataxias are rare cerebellar disorders. Population level mortality patterns in the United States in the previous decades remain insufficiently described. We quantified national mortality trends associated with hereditary ataxias and described stratified trends by sex, race, census region, and urbanization. We analyzed U.S. Multiple Cause of Death data from CDC WONDER for 2000 to 2020. Decedents were identified using ICD-10 code G11 for hereditary ataxias. Age-adjusted mortality rates (AAMRs) per 100,000 were computed using the 2000 U.S. standard population, with 95% confidence intervals. Temporal trends were evaluated using joinpoint regression to estimate annual percent change (APC). Forecasts through 2050 were generated using ARIMA, with sensitivity analysis using linear regression. National AAMR increased significantly from 2000 to 2020. Rising rates were observed in both sexes, with consistently higher mortality among males. The largest relative increases occurred among Black or African American individuals and in the West census region. Trends were directionally consistent across urbanization categories, and the forecast analyses also indicated increasing overall mortality. Sensitivity analyses produced qualitatively similar patterns, with wider uncertainty within strata. Mortality associated with hereditary ataxias increased in the United States between 2000 and 2020, highlighting growing clinical and public health needs related to cerebellar disease. Improved phenotyping and coding, earlier diagnosis, and registry-linked surveillance may refine estimates and support service planning.

**Graphical Abstract:**

**Figure:**
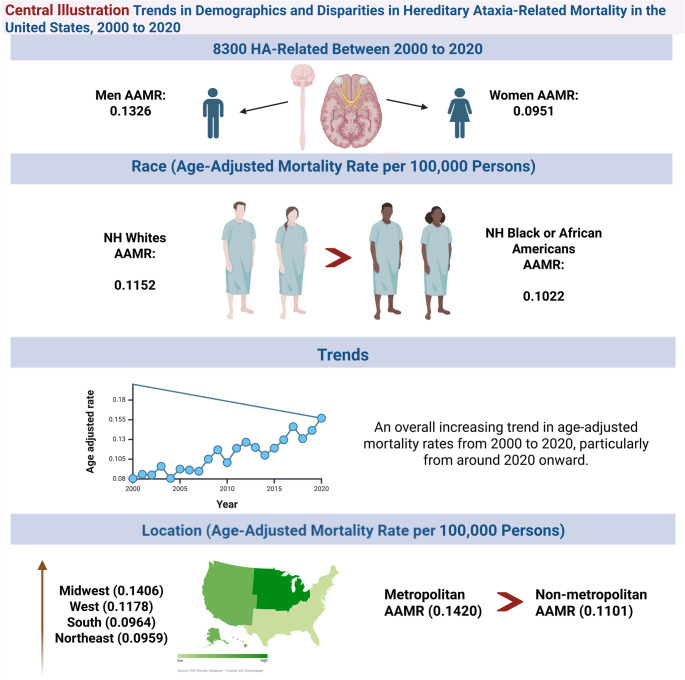
Mortality associated with hereditary ataxias increased in the United States from 2000 to 2020. National AAMR rose significantly over 2000 to 2020. Increases occurred in both sexes, with consistently higher rates among males. The largest relative increases were observed among Black or African American individuals and in the West census region. Patterns were directionally consistent across urbanization categories, and the forecast analysis also showed increasing mortality

**Supplementary Information:**

The online version contains supplementary material available at 10.1007/s12311-026-02046-7.

## Introduction

Hereditary ataxias (HAs) comprise a phenotypically and genetically heterogeneous set of progressive neurodegenerative disorders in which cerebellar and brainstem dysfunction leads to gait and limb incoordination, dysarthria, and abnormal eye movements [1, 2]. Recent epidemiologic syntheses estimate the overall prevalence of autosomal dominant spinocerebellar ataxias (SCAs) at approximately 5.6 per 100,000 [3]. Autosomal recessive conditions, including Friedreich ataxia and ataxia telangiectasia, typically present in childhood or adolescence and remain uncommon at the population level, although prevalence varies by ancestry and geography. Friedreich ataxia is the most prevalent inherited ataxia among individuals of European ancestry, with reported prevalence generally around 3 to 4 per 100,000 across many cohorts [4, 5], whereas ataxia telangiectasia is usually estimated at roughly 1 in 40,000 to 1 in 100,000 live births [6].

Despite their low prevalence, hereditary ataxias are associated with substantial morbidity and early mortality. In FRDA, hypertrophic cardiomyopathy and arrhythmias commonly accompany neurological deterioration. These cardiac complications are major contributors to premature death, and survival is strongly shaped by cardiac involvement and genetic severity [7–11]. Several autosomal recessive forms, including ataxia with vitamin E deficiency, cerebrotendinous xanthomatosis, Refsum disease, and primary coenzyme Q₁₀ deficiency, can be treated with dietary or biochemical replacement, but most hereditary ataxias still lack disease-modifying therapies [12, 13]. As a result, symptom-focused rehabilitation remains central to management, and research continues to face challenges related to small cohorts and slowly progressive trial endpoints [14]. One notable advance is the NRF2 activator omaveloxolone, the first agent shown to slow neurological decline in FRDA [9]. However, historical mortality analyses remain limited. For example, Spanish national data from 1981 to 2008 reported rising hereditary ataxia mortality, higher rates in males, and geographic clustering, demonstrating the value of death-certificate surveillance for assessing rare disease burden at scale [15].

Despite therapeutic advances, survival remains limited. In the EUROSCA cohort, 10-year survival was 57% for SCA1 and 73% to 87% for SCA2, SCA3, and SCA6 [16]. n addition, a prospective registry including 631 individuals with FRDA reported a cumulative 10-year survival of 87%, and found that arrhythmia, advanced disability, and diabetes independently increased mortality risk by approximately two fold to three fold [10]. These estimates highlight the need for population-based surveillance that goes beyond referral bias and small sample sizes. The U.S. Centers for Disease Control and Prevention’s Wide-ranging Online Data for Epidemiologic Research (CDC WONDER) Multiple Cause of Death database is publicly available and provides county-level counts and age-adjusted rates for deaths since 1999, coded using ICD-10, including hereditary ataxias (G11.0–G11.9), with stratification by age, sex, race or ethnicity, and geography [17]. These data therefore provide a distinct opportunity to quantify national mortality patterns and to benchmark the population-level impact of emerging interventions.

Using CDC WONDER, we report a 21-year analysis (2000 to 2020) of U.S. mortality attributable to hereditary ataxias, characterizing temporal trends, age-adjusted death rates, and demographic and regional disparities. We also applied autoregressive integrated moving average (ARIMA) models to project rates through 2050, in line with established public health time-series approaches to mortality forecasting [18, 19]. Establishing this population-level baseline is important for contextualizing clinical trial outcomes, identifying higher-risk subgroups, and informing future public health and therapeutic strategies.

## Materials and methods

This study was registered on the Open Science Framework (OSF) on 23 June 2025 (10.17605/OSF.IO/PRD4U).

### Study Setting

Death certificate data were obtained from the publicly available CDC WONDER Multiple Cause of Death (MCD) database [17]. The dataset includes all deaths registered in the 50 U.S. states and the District of Columbia from 2000 to 2020. We limited the analytic period to 2000 to 2020 because 1999 contained unreliable information for several variables.

### Study Design and Population

Deaths in which a hereditary ataxia was listed as the underlying cause of death (UCOD) or as a contributing cause were identified using ICD-10 code G11, which captures all subtypes within this classification. In the CDC WONDER query interface, the underlying cause of death field was left as “All causes of death,” while G11 (Hereditary ataxia) was entered in the multiple cause of death field, thereby capturing deaths in which hereditary ataxia was recorded anywhere on the death certificate, either as the underlying cause or as a contributing condition. Data were stratified by sex (male, female), race (Black or African American, White), census region (Northeast, Midwest, South, West), and urbanization (Large Central Metro, Large Fringe Metro, Medium Metro, Small Metro, Micropolitan, NonCore). All available data points from 2000 to 2020 were included. Data were extracted in June 2025 and initially imported into Microsoft Excel. Joinpoint regression was used to identify significant temporal trends and to estimate annual percent change (APC). Results were reported as age-adjusted mortality rates (AAMR) per the 2000 U.S. standard population, with 95% confidence intervals (CI), and statistical significance was defined as *p* < 0.05. Exact CDC WONDER query parameters were given in Supplementary File 5.

### Statistical analysis

#### Descriptive trend analysis

To assess temporal patterns in hereditary ataxia related mortality, we calculated age-adjusted mortality rates (AAMRs) per 100,000 population with 95% confidence interval (CI). Trends were evaluated using the National Cancer Institute’s Joinpoint Regression Program (version 4.9.0.0) to estimate annual percent change (APC) in AAMR with 95% CI [20]. The software fits log-linear regression models to the AAMR time series data, connecting successive straight-line segments at identified “joinpoints.” Each joinpoint represents a calendar year in which the rate of change shifts significantly. For each segment, we derived the APC and its 95% CI. An APC was classified as increasing or decreasing when the slope differed significantly from zero based on a two-tailed t test. Statistical significance was defined as *p* < 0.05 throughout.

#### Forecast modelling for AAMR from 2021 to 2050

An optimal ARIMA model was determined using the auto ARIMA function based on the Bayesian Information Criterion (BIC) and was then fitted to the data. The residuals of the model were assessed for white noise using the Ljung–Box test [21]. Visual trend identification and Augmented Dickey-Fuller tests indicated non-stationarity data (all *P* > 0.10), forecasts were generated with ARIMA models. The auto arima function (pmdarima 2.0) employed step wise AICc selection with limits p, q ≤ 3 and d ≤ 2 to prevent overfitting; seasonal terms were disabled (annual data). The Overall ARIMA (0,1,1) Male ARIMA (0,1,1), Female ARIMA (2,2,1), Black ARIMA (2,2,1), and White ARIMA (1,2,4), Northeast ARIMA (1,2,2), Midwest ARIMA (0,1,1), South ARIMA (2,1,2), West ARIMA (0,2,2), Large Central Metro (2, 2, 1), Large Fringe Metro (0, 2, 2), Medium Metro (0, 2, 2), Small Metro (0, 1, 1), Micropolitan (1, 2, 1), Non-core (0, 2, 2) were used based upon respective data. Thirty years ahead steps (2021–2050) were produced. Medians and two-sided 95% prediction intervals (PI) were derived from the parametric distribution of future innovations. Sensitivity analysis was also performed that compared our ARIMA forecasts with those from a simple linear regression (OLS) trend benchmark. The results of linear regression for all the strata are shown in Supplementary Fig. S6–S10 online. All analyses used Python 3.11 (Pandas 2.2, NumPy 1.26, SciPy 1.12, Statsmodels 0.14, pmdarima 2.0) within PyCharm 2024.1. Figures were produced with Matplotlib 3.9. To assess short-term forecast validity, ARIMA models fit on the 2000 to 2020 series were compared with newly available observed CDC WONDER age-adjusted mortality rates for 2021 to 2024. Validation was performed for the overall, sex-stratified, and census region-stratified series by comparing forecasted and observed AAMRs and calculating absolute prediction error. For the overall series, coverage of the 95% prediction intervals was also examined.

### Standard protocol approvals, registrations, and patient consents

This study was considered exempt from institutional review board approval because it used deidentified, publicly available government data and was conducted and reported in accordance with the STROBE (Strengthening the Reporting of Observational Studies in Epidemiology) guidelines. Data points suppressed in the CDC WONDER database for confidentiality reasons were excluded from analysis.

## Results

**Fig. 1:**
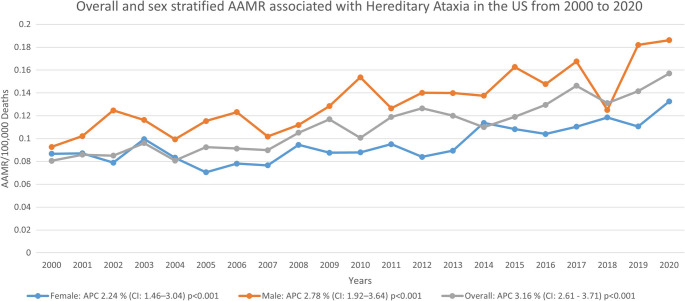
Overall and Sex-stratified Hereditary Ataxia Related AAMRs per 100,000 in the United States, from 2000–2020

**Fig. 2:**
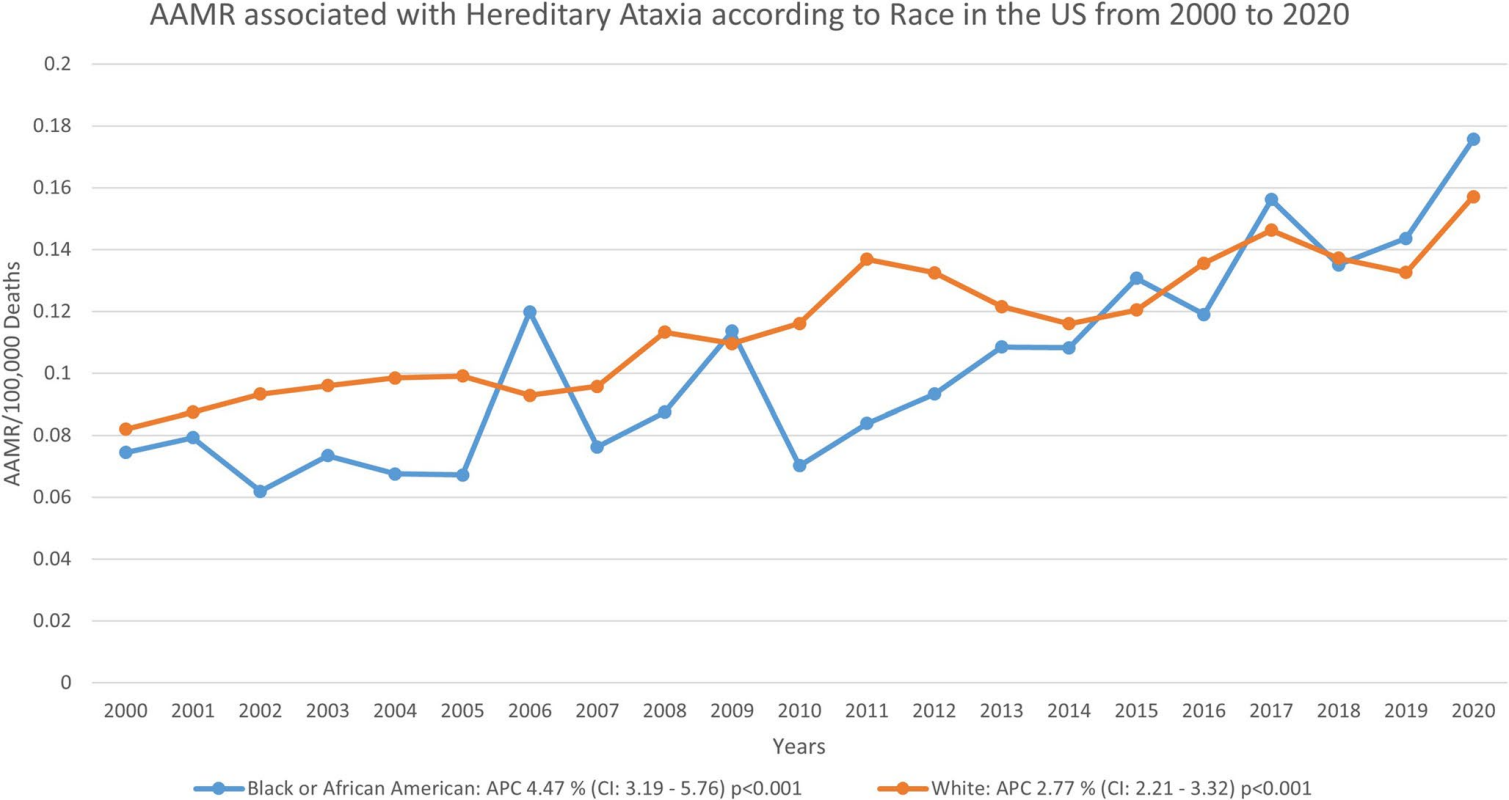
Hereditary Ataxia Related AAMRs per 100,000 Stratified by Race or Ethnic Groups in the United States, from 2000–2020

**Fig. 3:**
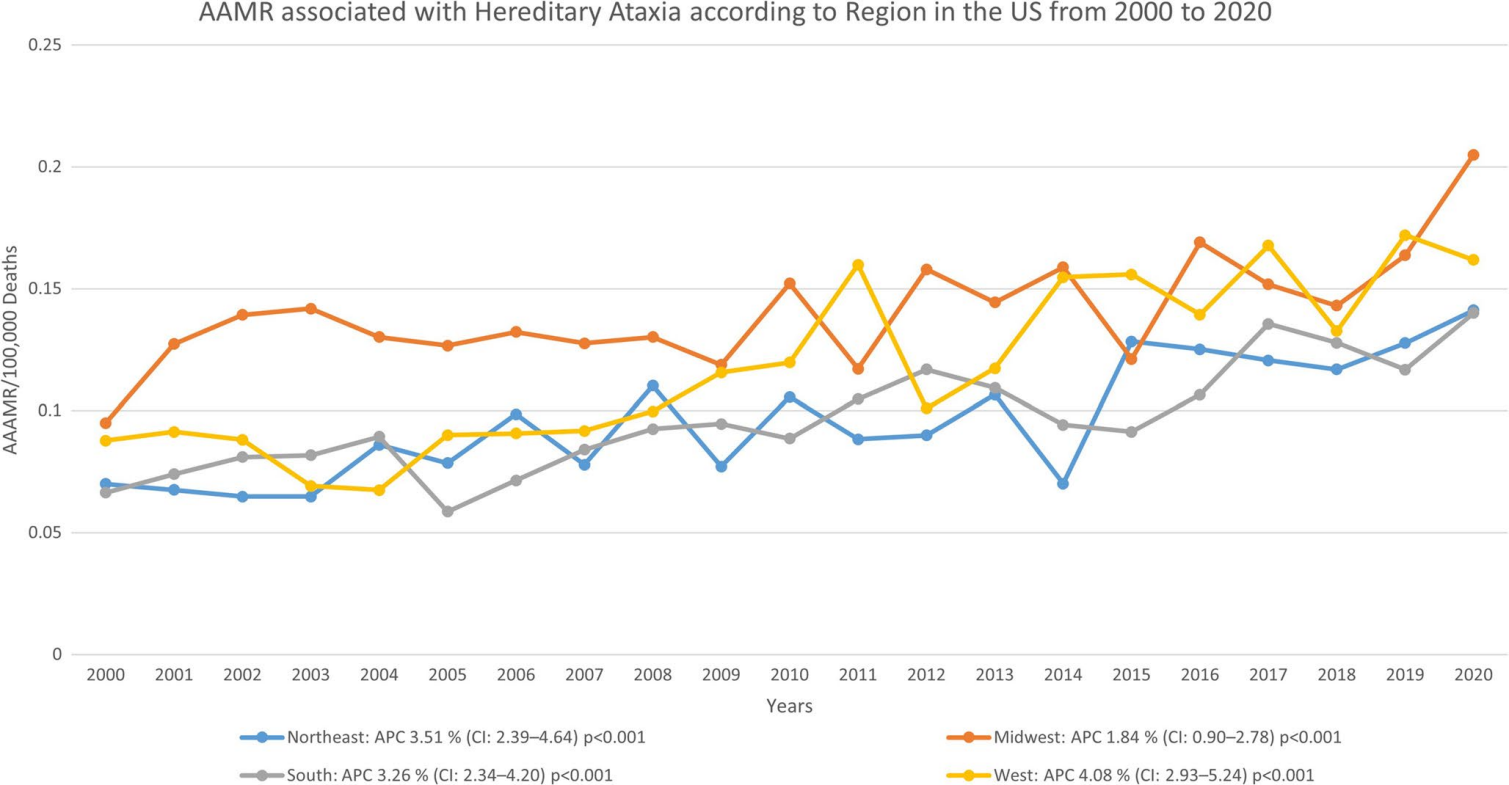
Hereditary Ataxia Related AAMRs per 100,000 Stratified by Census Region in the United States, from 2000–2020

According to Multiple Cause of Death (MCOD) dataset (all ages combined), 8,039 people with hereditary ataxia died from 2000 to 2020 in United States. A steady rise was observed across these years with a prominent increase from 2015 to 2020. The age-adjusted mortality rate (AAMR) of hereditary ataxias showed a significant increase from 0.0805 in 2000 to 0.157 in 2020 (APC: 3.16; 95% CI: 2.61 to 3.71). Trends were further evaluated by sex, race, and census region (Figs. 1, 2 and 3), and APC estimates are summarized in Table 1. Yearly AAMRs and the underlying extracted data are provided in Supplementary Files S1 and S2. Urbanization-stratified trends are shown in Supplementary Figure S1.

**Table 1:** Annual Percentage Changes (APCs) in Hereditary Ataxia Mortality in the USA from 2000 to 2020

Variable	Lower Endpoint	Upper Endpoint	APC (95% CI)	*P*-Value
Overall	2000	2020	3.16% (2.61–3.71)	< 0.001
Sex				
Male	2000	2020	2.78% (1.92–3.64)	< 0.001
Female	2000	2020	2.24% (1.46–3.04)	< 0.001
US Census Region				
Northeast	2000	2020	3.51% (2.39–4.64)	< 0.001
Midwest	2000	2020	1.84% (0.90–2.78)	< 0.001
South	2000	2020	3.26% (2.34–4.20)	< 0.001
West	2000	2020	4.08% (2.93–5.24)	< 0.001
Race				
Black/African American	2000	2020	4.47% (3.19–5.76)	< 0.001
White	2000	2020	2.77% (2.21–3.32)	< 0.001
Urbanization				
Large Central Metro	2000	2020	4.49% (CI: 3.16–5.82)	< 0.001
Large Fringe Metro	2000	2020	3.35% (CI: 2.34–4.36)	< 0.001
Medium Metro	2000	2020	3.16% (CI: 2.02–4.31)	< 0.001
Small Metro	2000	2020	2.41% (CI: 1.13–3.71)	< 0.001
Micropolitan (Nonmetro)	2000	2020	2.41% (1.35–3.49)	< 0.001
Non-core (Nonmetro)	2000	2020	2.15% (CI: 0.41–3.92)	0.018

### Gender

Sex stratified analysis showed increased AAMRs in both males and females from 2000 to 2020 where males had shown higher AAMR than the females. Females showed significant increase in AAMR from 0.0867 in 2000 to 0.1325 in 2020 (APC: 2.24; 95% CI: 1.46 to 3.04). Similarly, males also reported rise in AAMR over time from 0.0927 in 2000 to 0.1862 in 2020 (APC: 2.78; 95% CI: 1.92 to 3.64), as shown in Fig. 1. In descriptive between-group comparisons, male AAMR remained higher than female AAMR throughout the study period, with the absolute male-female difference widening from 0.0060 per 100,000 in 2000 to 0.0537 per 100,000 in 2020, and the male-to-female rate ratio increasing from 1.07 to 1.41.

### Race

Race stratified analysis also showed increased AAMRs in both White individuals as well as Black or African American individuals from 2000 to 2020. In early years, AAMRs were initially higher among White individuals, but in later years the AAMR in Black or African American population surpassed them. White individuals showed significant increase in AAMR from 0.0820 in 2000 to 0.1571 in 2020 (APC: 2.77; 95% CI: 2.21 to 3.32). Similarly, Black or African American individuals also reported rise in AAMR over time from 0.0745 in 2000 to 0.1757 in 2020 (APC: 4.47; 95% CI: 3.19 to 5.76), as shown in Fig. 2. In descriptive comparisons by race, White AAMR was slightly higher than Black or African American AAMR in 2000, with an absolute difference of 0.0075 per 100,000 and a rate ratio of 1.10; however, by 2020, Black or African American AAMR exceeded White AAMR, with an absolute difference of 0.0186 per 100,000 and a rate ratio of 1.12, indicating a reversal in the relative burden over time.

### Region

**Fig. 4:**
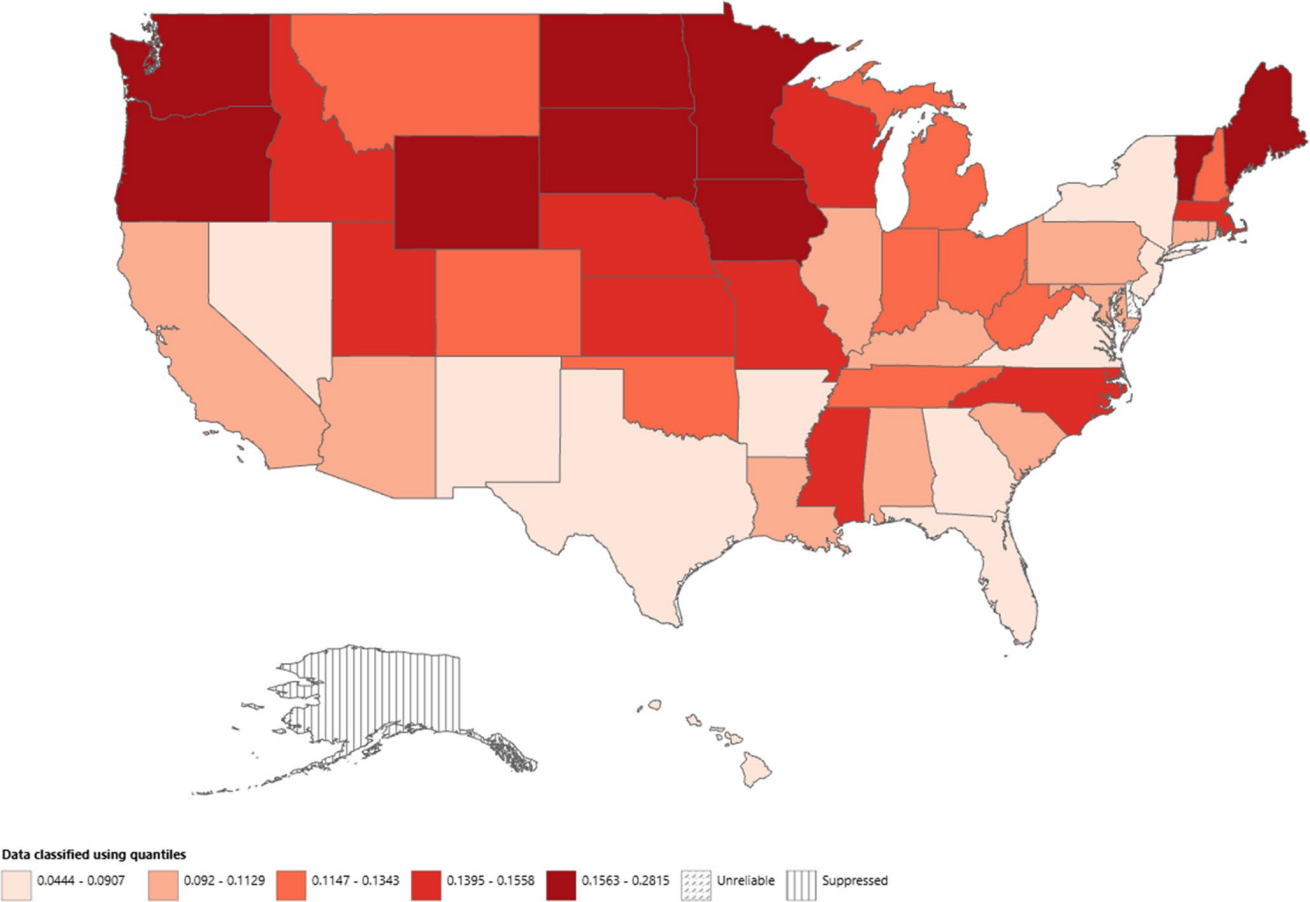
Hereditary Ataxia Related AAMR Stratified by States in the United States, from 2000–2020

In all U.S. census regions, AAMR increased significantly from 2000 to 2020. The Midwest and West reported highest AAMR rates. In the Northeast, AAMR increased from 0.07 in 2000 to 0.1411 in 2020 (APC: 3.51; 95% CI: 2.39 to 4.64). In the Midwest, AAMR increased from 0.0949 in 2000 to 0.205 in 2020 (APC: 1.84; 95% CI: 0.90 to 2.78). In the South, AAMR increased from 0.0664 in 2000 to 0.14 in 2020 (APC: 3.26; 95% CI: 2.34 to 4.20). In the West, AAMR increased from 0.0878 in 2000 to 0.1618 in 2020 (APC: 4.08; 95% CI: 2.93 to 5.24), as shown in Fig. 3. State-level AAMRs for 2000 to 2020 are presented in Fig. 4. In descriptive regional comparisons, the Midwest had the highest AAMR and the South the lowest at both time points examined. The absolute regional difference widened from 0.0285 per 100,000 in 2000 to 0.0650 per 100,000 in 2020, while the highest-to-lowest regional rate ratio increased modestly from 1.43 to 1.46. Although the Midwest had the highest absolute AAMR by 2020, the West showed the steepest relative increase over time, consistent with its highest APC.

### ARIMA based forecast of AAMRs from 2021 to 2050

**Fig. 5:**
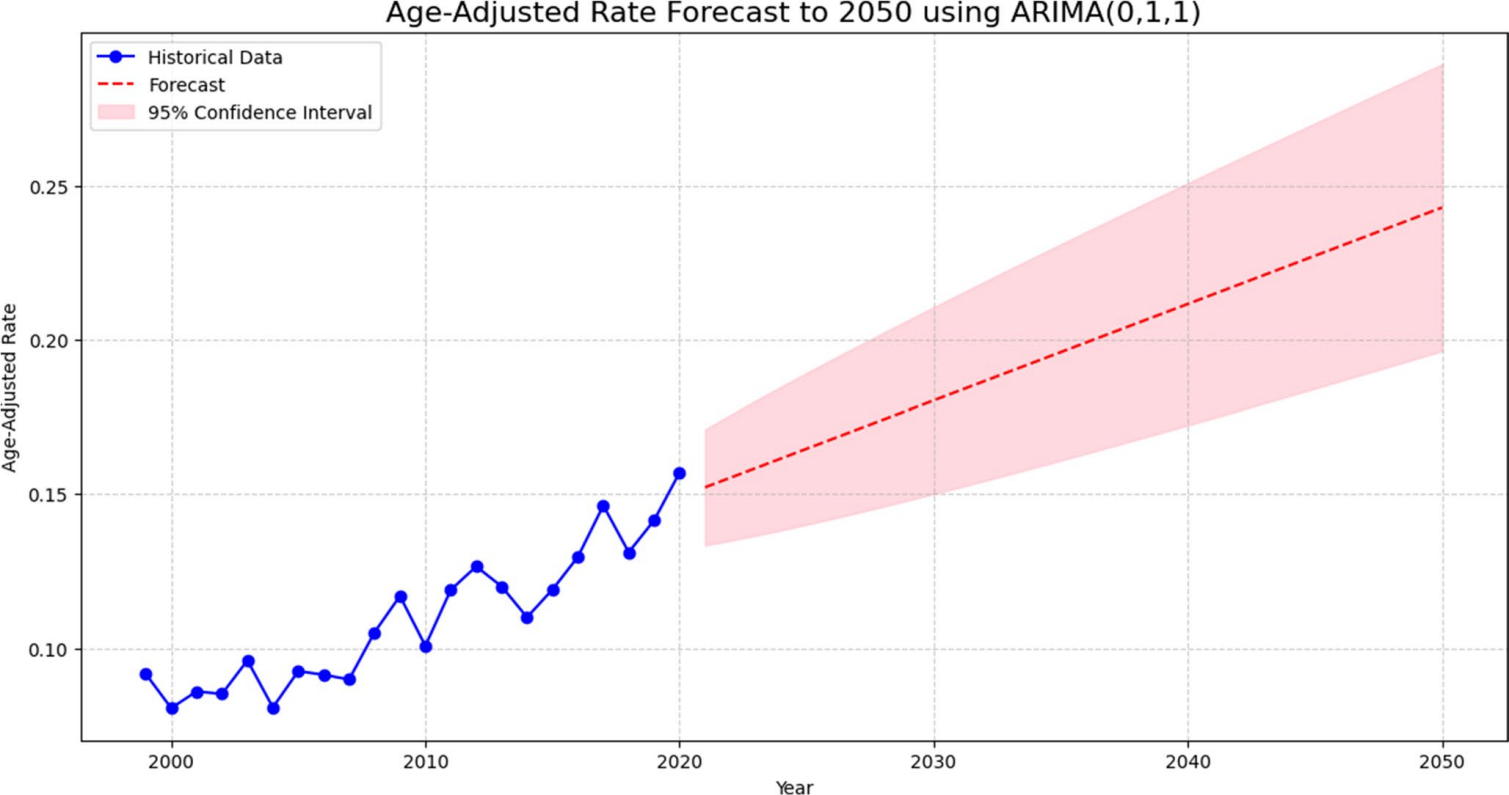
ARIMA Modelling Based Forecast of Overall Hereditary Ataxia Related AAMRs per 100,000 in the United States, from 2021 to 2050

All strata were modeled using univariate auto ARIMA. Results are presented as median projections with 95% prediction intervals (PIs). The overall AAMR was best fit by an ARIMA (0, 1, 1) model and met all diagnostic criteria. Using the observed 2020 rate of 0.157 per 100,000 as the starting point, the model projects an increase to 0.196 (PI: 0.161 to 0.231) in 2035 and to 0.243 (PI: 0.196 to 0.289) in 2050. Forecasts indicated a continued rise in AAMRs through 2050 as shown in Fig. 5. Forecasts stratified by sex, race, census region, and urbanization are provided in Supplementary Figures S2 to S5, and ARIMA specifications with yearly forecast values are detailed in Supplementary File S2. Sensitivity analyses using linear regression yielded median projections within ± 5% of the ARIMA medians across all strata but produced narrower 95% PIs that were unchanged for most strata (Supplementary Figures S6 to S10), suggesting that the auto ARIMA approach captures additional uncertainty inherent to rare-event time series within each stratum. To assess short-term forecast performance, predicted AAMRs from models trained on the 2000 to 2020 data were compared with observed CDC WONDER rates for 2021 to 2024. For the overall series, forecasted AAMRs were 0.1522, 0.1553, 0.1585, and 0.1616 per 100,000 for 2021, 2022, 2023, and 2024, respectively, compared with observed values of 0.1432, 0.1581, 0.1601, and 0.1749. The mean absolute error was 0.0067, and all observed values fell within the corresponding 95% prediction intervals. Validation of sex-stratified and region-stratified forecasts showed similar overall directional agreement but greater year-to-year deviation, particularly in smaller strata, consistent with the higher variability expected in rare-event subgroup series. The data for overall mortality from 2021 to 2024 has been uploaded in Supplementary File S3 while exact CDC WONDER query has been uploaded in S4.

## Discussion

Hereditary ataxias comprises of many different and diverse types of neurodegenerative disorders that are rare but collectively have contributed to global disease burden [22]. Life expectancy varies widely: SCA6 patients have 10-year survival of 87%, whereas SCA1 patients fall to 57% over the same interval [16]. Death is seldom the direct result of cerebellar failure; instead, aspiration pneumonia, sepsis, or cardiomyopathy claim most lives [23]. Dysphagia interventions, including compensatory swallowing therapy and prophylactic tube feeding, improve nutritional status but do not abolish risk, 30 day post-gastrostomy mortality still approaches 11% in advanced ataxia cohorts [24, 25]. Recent physiologic studies confirm profound cough impairment in cerebellar disease, explaining the high prevalence of lower airway infection [26]. This is a CDC based retrospective study which analyzes the mortality rates from 2000 to 2020. This study observed that the mortality from hereditary ataxias is continuously rising significantly in these years. In United States, it has been recognized as a cause of premature deaths over the last two decades as the overall age-adjusted mortality rate (AAMR) increased from 0.0805 in 2000 to 0.157 in 2020 (APC: 3.16%, CI: 2.61–3.71), and an acceleration after 2015. These findings extend and update earlier evidence. Leone and colleagues, using U.S. death certificates from 1971 to 1973 to 1978, reported higher age-adjusted mortality in men and whites with frequent cardiac causes recorded [27], while our analysis shows similar rise in men also, constant significant increases across all strata and a marked accelerated rise late after 2015 in overall population.

This upward trajectory is similar to the 54% increase in deaths due to Alzheimer from 1999 to 2014 [28] and another study also reported same rise in mortality for the next 4 years until 2018 [29]. This upward trajectory also reported for multiple sclerosis in which mortality rise occurred from 1999 to 2018 and then a sharp increase in mortality until 2020 [30]. Similar trends for overall rise were observed for mortality related to arterial fibrillation and dementia [31], and Parkinson’s disease [32]. The only prior U.S. population study, based on 1970s ICD-8/9 data, reported a mortality rate of 0.33 but did not examine trends [27]. Our findings indicate a sustained increase in recorded hereditary ataxia mortality on U.S. death certificates over the study period. The persistent upward trend may reflect a combination of authentic disease burden and improved ascertainment, including broader access to molecular testing and higher diagnostic yields with next-generation sequencing panels and exome sequencing in suspected genetic ataxias [33]. Because we used multiple-cause coding, our approach aligns with CDC guidance for capturing both underlying and contributing causes, which improves sensitivity for rare disorders on death certificates.

In this study, it has been observed that males have a higher AAMR compared with females which is a key determinant showing sex-based disparity. Male mortality increased almost two times, rising from 0.0927 in 2000 to 0.1862 in 2020 (APC: 2.78%, CI: 1.92–3.64).), while female mortality increased from 0.0867 in 2000 to 0.1325 in 2020 (APC: 2.24%, CI: 1.46–3.04). Experimental studies in knockout mice demonstrate severe cardiac remodeling and earlier death in males than females, and human imaging studies report sex-linked differences in left ventricular geometry and function in Friedreich ataxia cohorts [34, 35]. Cardiac dysfunction accounts for most deaths in Friedreich ataxia cohorts, which supports higher male mortality if men experience greater cardiac involvement, although definitive population-level sex-specific cause-of-death partitions for all hereditary ataxias remain sparse [11]. Although many hereditary ataxias are autosomal, these data suggest that sex specific biological or health care factors may influence outcomes. The narrowing difference in APCs between sexes in our series warrants attention to potential diagnostic delays or care access barriers in women, but that interpretation should be tested in linked clinical datasets. There is further research needed in the future to assess these differences which can also be due to severity of disease, difference in diagnosis patterns, or other health conditions that can affect one sex more than the other.

Racial mortality patterns reversed over two decades. In early years, highest AAMR was observed in White individuals while by 2020 the AAMR became higher in the Black or African American population. Black/African Americans rate increased from 0.0745 in 2000 to 0.1757 in 2020 (APC: 4.47%, CI: 3.19–5.76), leaving behind the White rate, which increased from 0.082 in 2000 to 0.1571 in 2020 (APC: 2.77%, CI: 2.21–3.32). Black/African American rates surpassed white rates, with APC 4.47% in Black/African Americans versus 2.77% in Whites. Several mechanisms may contribute. Founder effects and subtype distributions vary by region and ancestry, which can shift observed mortality when ascertainment improves across diverse communities [36, 37]. In the 1970s, White mortality exceeded non-White mortality [27], indicating historical under ascertainment among minority populations. The increase in mortality in Blac/African Americans might be due to improved diagnostics over the years, broader access to genetics-based testing and rising awareness among the Black/African American communities who were underdiagnosed in early years. However, the steeper increase also indicates ongoing disparities in access to multidisciplinary care and supportive services [38]. It is also possible that in early years the Black or African Americans were not so aware of these disease patterns and less aware of these causes and disorders contributing to mortality rates that showed the lower mortality rates. Because we analysed MCOD rather than registry diagnoses, improved certification of hereditary ataxia as a contributing cause among African American decedents may also increase observed rates. These hypotheses should be evaluated with linked clinical, genetic, and vital records.

Geographic trends in all U.S. major census regions showed that AAMR was notably increased. The West experienced the fastest growth increased from 0.0878 in 2000 to 0.1618 in 2020 (APC: 4.08%, CI: 2.93–5.24), followed by the Northeast increased from 0.07 in 2000 to 0.1411 in 2020 (APC: 3.51%, CI: 2.39–4.64), South increased from 0.0664 in 2000 to 0.14 in 2020 (APC: 3.26%, CI: 2.34–4.20), and Midwest increased from 0.0949 in 2000 to 0.205 in 2020 (APC: 1.84%, CI: 0.90–2.78). Geographic structure is expected in hereditary ataxias because of founder effects and migration patterns. For instance, regional clustering has been documented in Spain for hereditary ataxia mortality and in the Americas for specific SCA founders, while SCA6 has been reported as one of the most common autosomal dominant cerebellar ataxias in the North of England. Such heterogeneity can affect regional mortality profiles when ascertainment improves or when subtype mixtures differ by region [15, 37, 39]. Regions with major academic and genomic centres may have seen more rapid recoding of cerebellar disorders into hereditary ataxia diagnoses, while areas with established ataxia clinics like the Midwest may have reached diagnostic saturation earlier. This could be due to better healthcare infrastructure over the years and improved health policies. However, it is not easy to compare the results of this study with previous studies as there is very less nationwide data present on hereditary ataxias.

These population trends when aligned with genotype specific studies clarifies the overall picture. Recessive early-onset forms such as ataxia telangiectasia maintain a median survival under 25 years [40], and Friedreich’s ataxia remains fatal by the late 30s despite improved cardiac care [11]. In contrast, Autosomal dominant spinocerebellar ataxias typically manifest in midlife and progress slowly, with median survival between 57 and 87 years [16]. As genetic recognition of both early and late onset subtypes has widened, more deaths have been appropriately coded under ICD-10 G11* which were categorized somewhere else before, showing an observed increase in mortality rate but in actual overall change in mortality rate has not really changed. This highlights the importance of early diagnosis and disease management in these individuals. These trends could be a result of three major factors. First, the genomic revolution after 2010 that has improved the diagnostic precision, shifting many deaths from nonspecific “cerebellar degeneration” codes into hereditary ataxia categories. Secondly, Despite progress in some subtypes, including FRDA, disease-modifying options remain unavailable for most hereditary ataxias. Experimental approaches, repeat-silencing oligonucleotides, AAV-mediated frataxin replacement, dentate-nucleus deep-brain stimulation, are promising but remain investigational [41–43]. So enhanced diagnosis did not translate into prolonged survival. Third, structural inequities in health care continue to influence who receives genetic testing and specialized management, effecting demographic and regional disparities.

Looking forward, the stratified Auto-ARIMA projections indicate that national hereditary-ataxia mortality will rise from 0.157 deaths/100,000 in 2020 to 0.24 by 2050 and sensitivity analyses with linear models producing similar medians. ARIMA methods are established for short to medium-term mortality and incidence forecasting in public health, including injury and communicable disease outcomes, and the use of differencing to achieve stationarity with moving average terms is common in monotonic mortality series [18, 44]. The short-term validation against observed 2021 to 2024 mortality rates supports reasonable performance of the overall model, although subgroup forecasts were less stable, reinforcing the need for caution when interpreting long-range projections in smaller strata. Because hereditary ataxias are rare and certification practices can evolve, wider prediction intervals from ARIMA are reasonable and likely capture uncertainty better than simple linear extrapolation. Therapeutic developments are unlikely to reverse population mortality in the near term.

### Limitations

Several limitations merit emphasis. Death-certificate data are susceptible to misclassification and underreporting of rare genetic conditions. The MCOD approach mitigates this by including both underlying and contributing causes, but it cannot disaggregate by genotype or age at onset. Changes in diagnostic practice and certifier awareness over time can influence observed trends. Nonetheless, national coverage, standardized age adjustment, and the internal consistency of increasing rates across sex, race, region, and urbanization provide strong evidence for a rising burden of hereditary ataxia mortality. Because the study used stratified aggregated death-certificate data from CDC WONDER, the observed differences by sex, race, and region are descriptive and unadjusted. These strata are not independent in the underlying population, and regional patterns may partly reflect differences in demographic composition. Accordingly, these findings should not be interpreted as mutually adjusted or causal associations.

Next steps include linkage of MCOD data to clinical registries and genetic databases to resolve subtype-specific risk, and equity-focused implementation research to improve access to genetic testing and multidisciplinary management in underserved communities, including rural areas. Together, these data argue for sustained surveillance, targeted referral pathways to ataxia centers, and trials that incorporate cardiac outcomes in Friedreich ataxia given the central role of cardiomyopathy in mortality. The inclusion of 2020 also merits caution. The COVID-19 pandemic substantially altered U.S. mortality patterns and may have affected both care pathways and death-certificate coding practices. Accordingly, the 2020 endpoint may partly reflect pandemic-related system effects rather than hereditary ataxia-specific changes alone. However, short-term validation against newly observed 2021 to 2024 CDC WONDER data showed that the overall forecast remained reasonably aligned with post-2020 rates, suggesting that the increasing trajectory was not solely an artifact of the pandemic year. Because our analysis is based on death-certificate data, it cannot disentangle direct hereditary ataxia-related mortality from indirect pandemic effects such as disrupted access to care, changes in certification, or competing causes of death.

## Conclusion

Mortality due to hereditary ataxias in the United States increased from 0.0805 to 0.157 per 100,000 between 2000 and 2020, an annual percentage change of 3.16%. Increases were steeper among men, Black or African American populations, and residents of the West, while the Midwest reached the highest AAMR by 2020. These patterns may reflect improved diagnostic recognition, minimal therapeutic progress at the population level, and persistent disparities in access to specialty care. Limitations include potential misclassification or underreporting on death certificates even in multiple cause analyses, lack of genotype and age at onset data, secular changes in coding and diagnostic practice, and small counts in some strata that widen uncertainty. These findings underscore the need for timely diagnosis, coordinated multidisciplinary management, and equitable access to services. Priorities include ataxia focused clinics, telemedicine for high burden regions, culturally tailored outreach across racial communities, and coverage for genetic testing and counselling. Clinical programs should integrate cardiac surveillance for Friedreich ataxia, rehabilitation, and palliative support, and should track quality metrics such as time to diagnosis, adherence to guideline-based care, and retention in follow up. By providing the first stratified national forecast, with an overall AAMR projected at 0.243 in 2050, this study offers a quantitative baseline for planning and a benchmark to measure the impact of targeted interventions, while recognizing that forecasts remain sensitive to model assumptions and future changes in therapy and certification practices.

## Supplementary Information

Below is the link to the electronic supplementary material.

Supplementary Figure 1 (JPG 572 KB)

Supplementary Figure 2 (PNG 97.9 KB)

Supplementary Figure 3 (PNG 79.5 KB)

Supplementary Figure 4 (PNG 177 KB)

Supplementary Figure 5 (PNG 240 KB)

Supplementary Figure 6 (PNG 73.9 KB)

Supplementary Figure 7 (JPG 267 KB)

Supplementary Figure 8 (JPG 572 KB)

Supplementary Figure 9 (JPG 566 KB)

Supplementary Figure 10 (JPG 574 KB)

Supplementary File 1 (DOCX 28.5 KB)

Supplementary File 2 (DOCX 35.9 KB)

Supplementary File 3 (DOCX 15.5 KB)

Supplementary File 4 (DOCX 16.1 KB)

## Data Availability

Analytic code was executed in Jupyter Notebook and is available from the corresponding author on reasonable request.

## Declarations

### Supplementary data and materials

Aggregated mortality data are available from CDC WONDER. The processed dataset and study materials supporting the conclusions of this article are available in Supplementary Files online.
