# Supplementary figures and images for: Trends and Disparities in Mortality from Hereditary Ataxia in United States, 2000–2020: A Retrospective Analysis with Projections to 2050
Source: Cerebellum. 2026 Jun 29;25(4):101. doi: 10.1007/s12311-026-02046-7 (PMC13314688; doi:10.1007/s12311-026-02046-7)

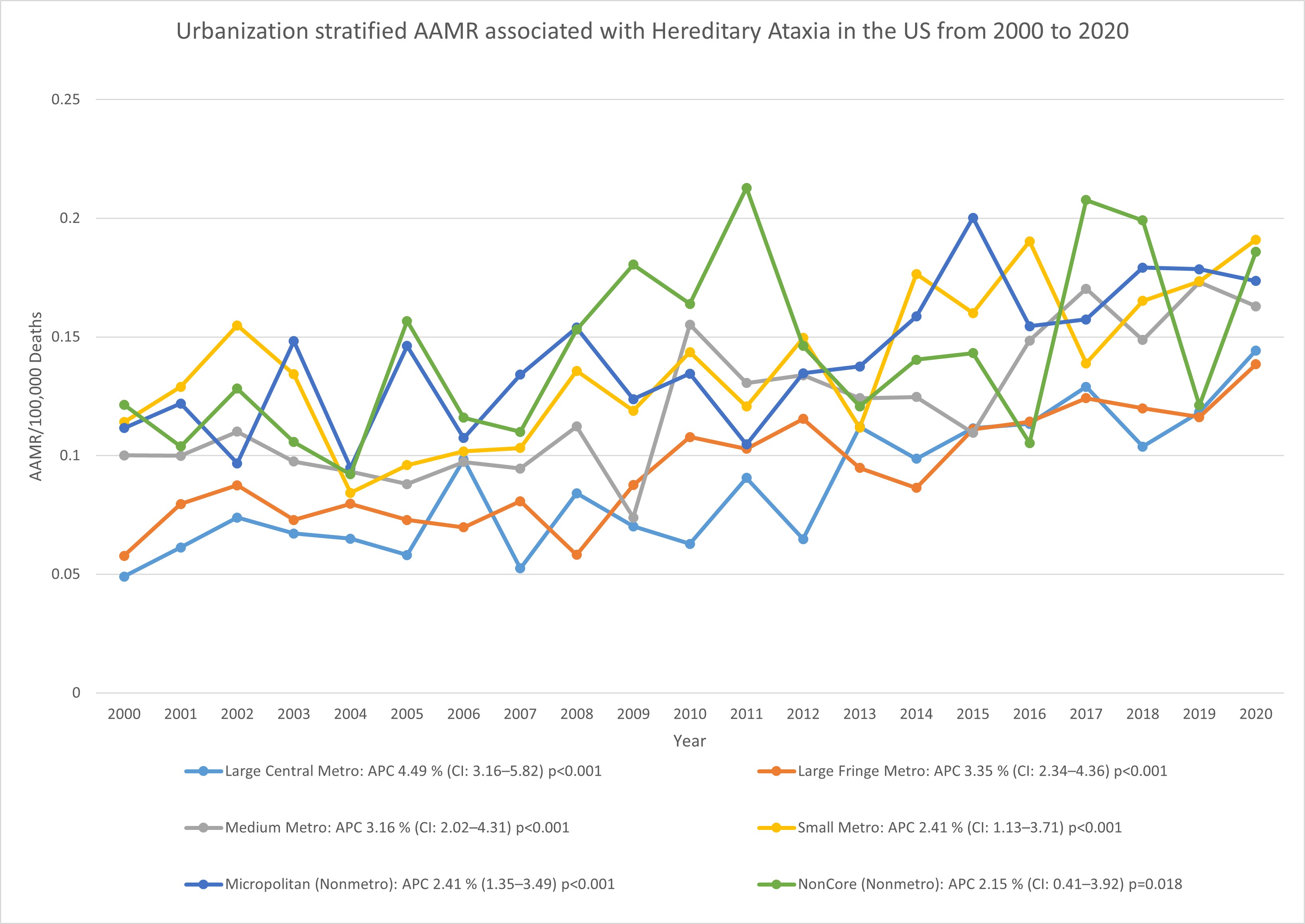

Supplement: Supplementary file 1 — Supplementary Figure 1 (JPG 572 KB) [file 12311_2026_2046_MOESM1_ESM.jpg]

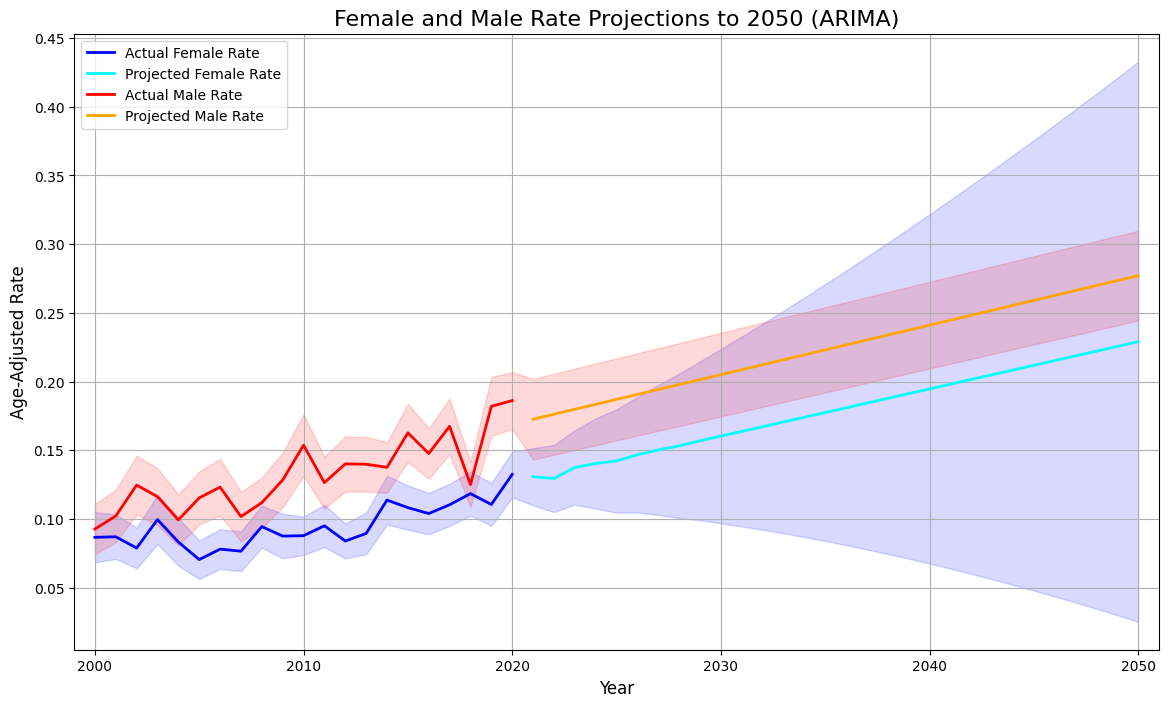

Supplement: Supplementary file 2 — Supplementary Figure 2 (PNG 97.9 KB) [file 12311_2026_2046_MOESM2_ESM.png]

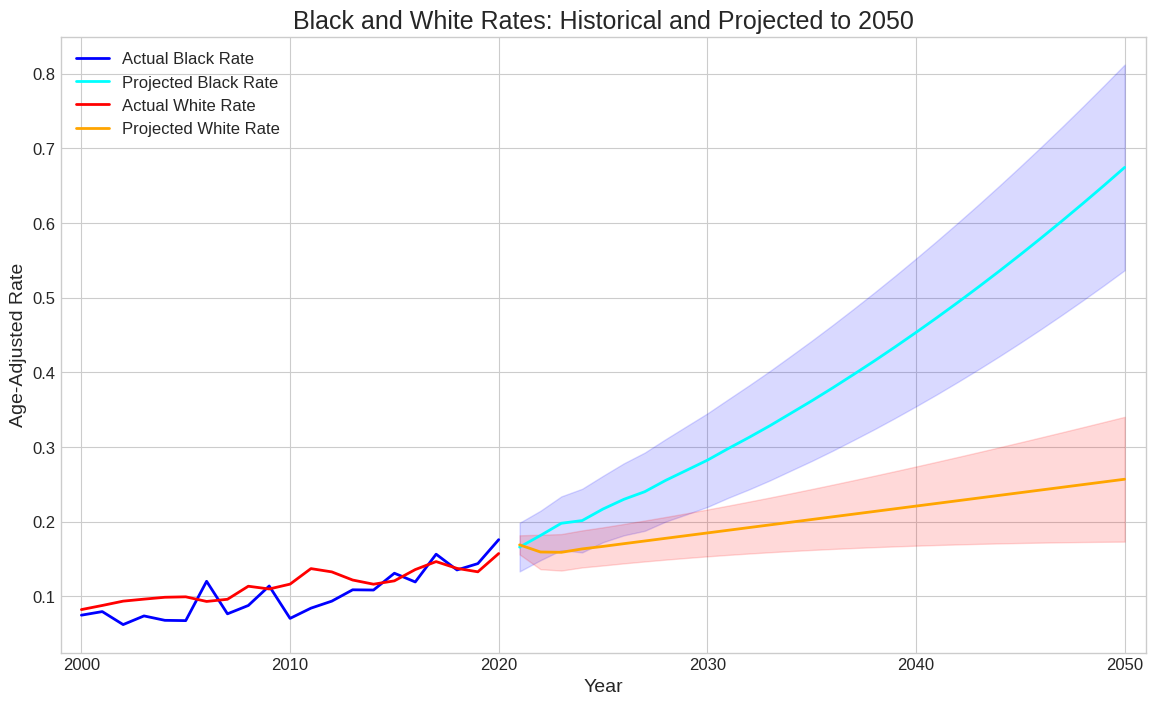

Supplement: Supplementary file 3 — Supplementary Figure 3 (PNG 79.5 KB) [file 12311_2026_2046_MOESM3_ESM.png]

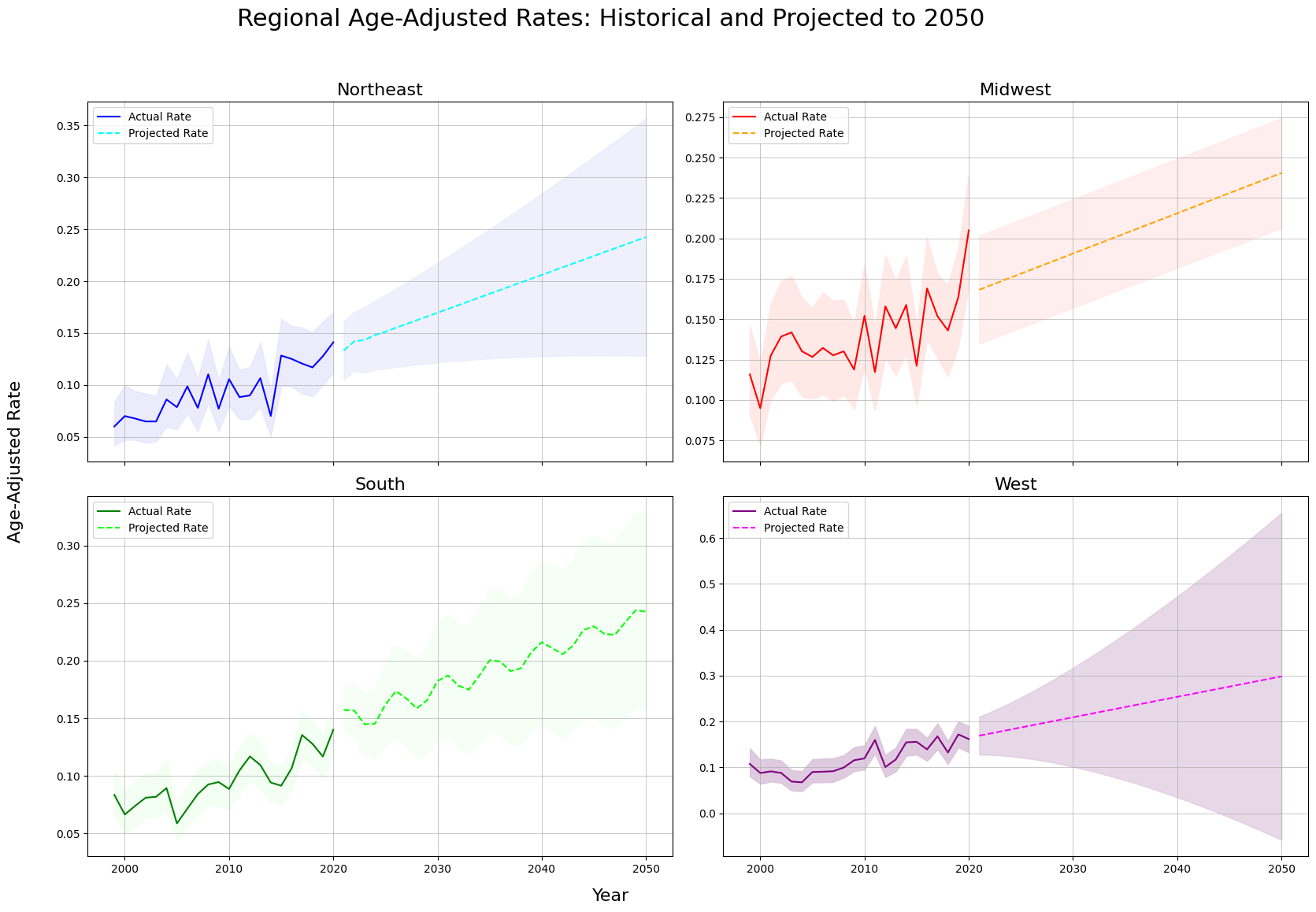

Supplement: Supplementary file 4 — Supplementary Figure 4 (PNG 177 KB) [file 12311_2026_2046_MOESM4_ESM.png]

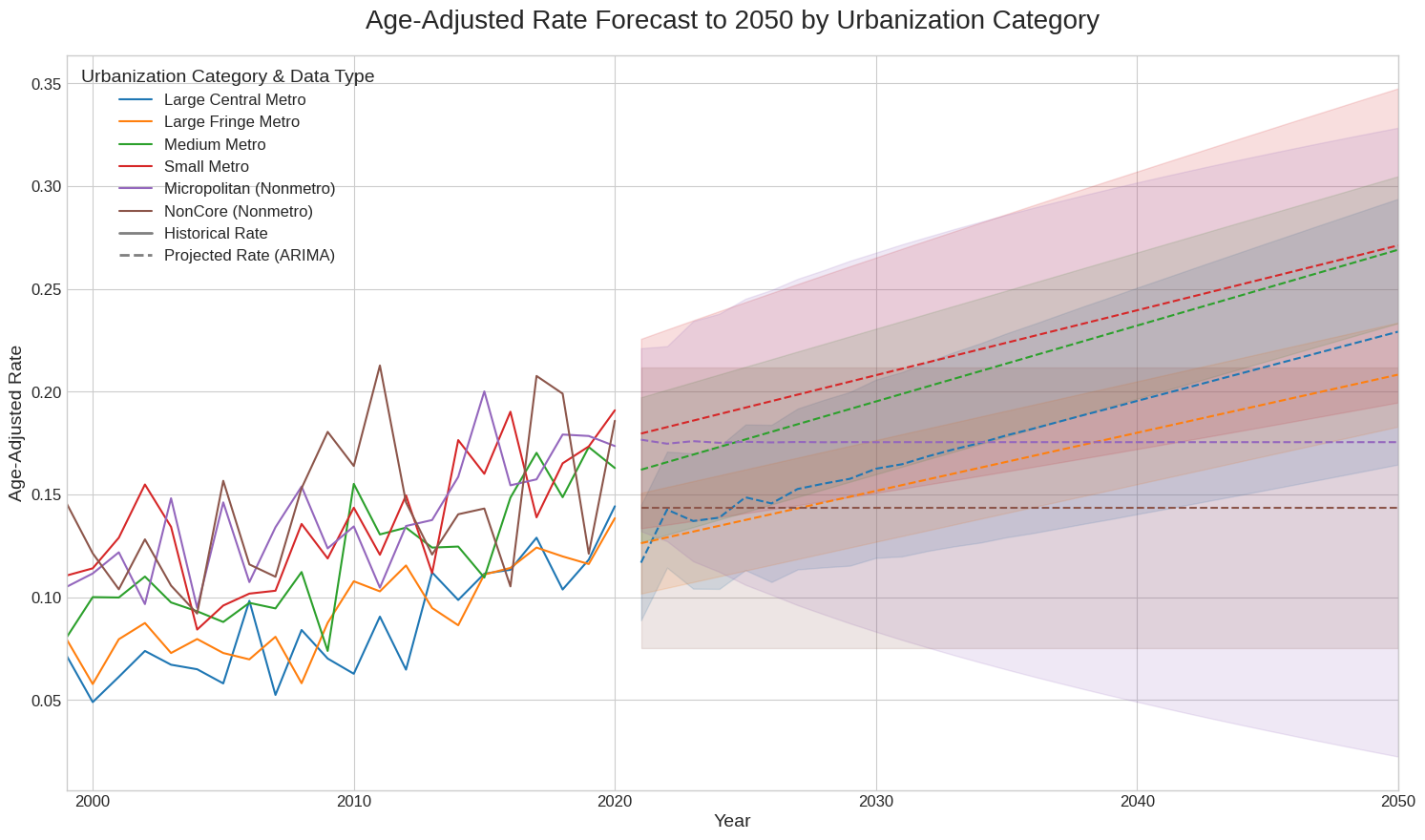

Supplement: Supplementary file 5 — Supplementary Figure 5 (PNG 240 KB) [file 12311_2026_2046_MOESM5_ESM.png]

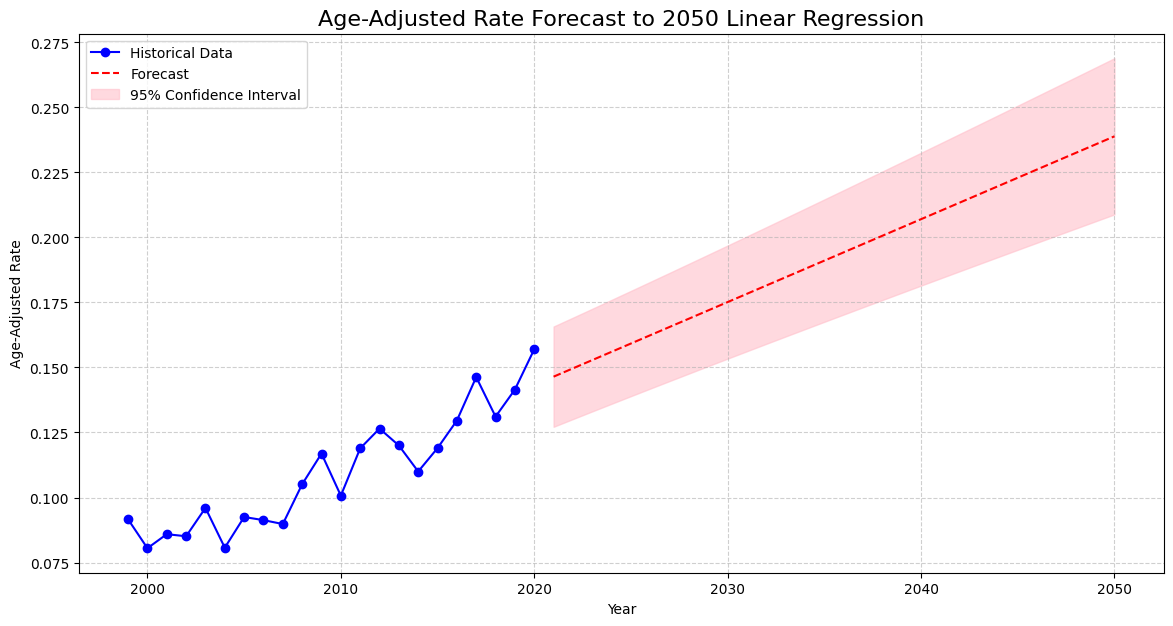

Supplement: Supplementary file 6 — Supplementary Figure 6 (PNG 73.9 KB) [file 12311_2026_2046_MOESM6_ESM.png]

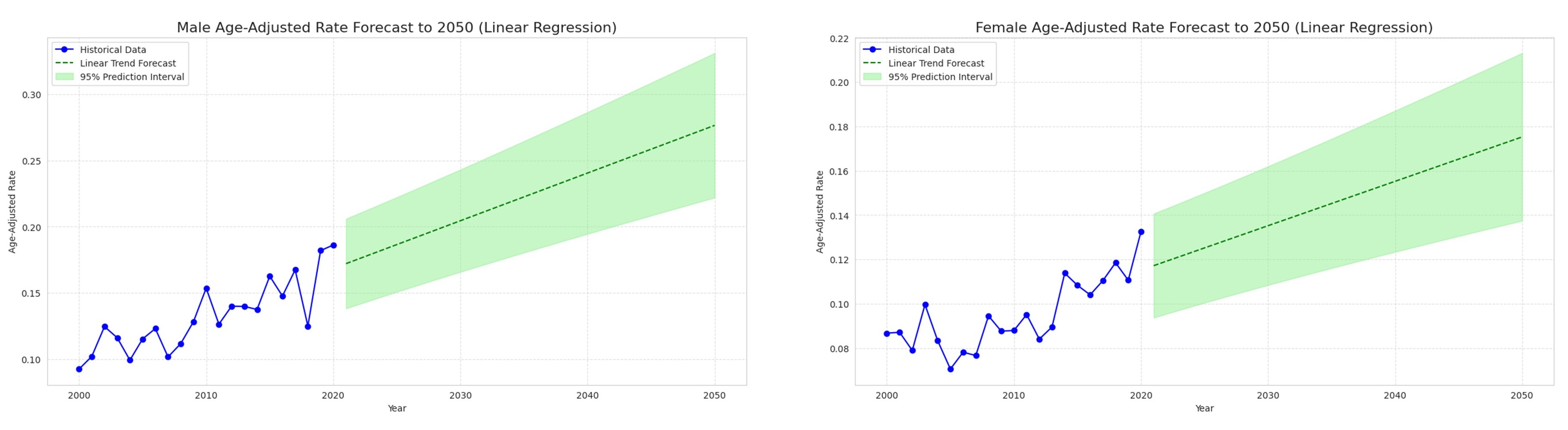

Supplement: Supplementary file 7 — Supplementary Figure 7 (JPG 267 KB) [file 12311_2026_2046_MOESM7_ESM.jpg]

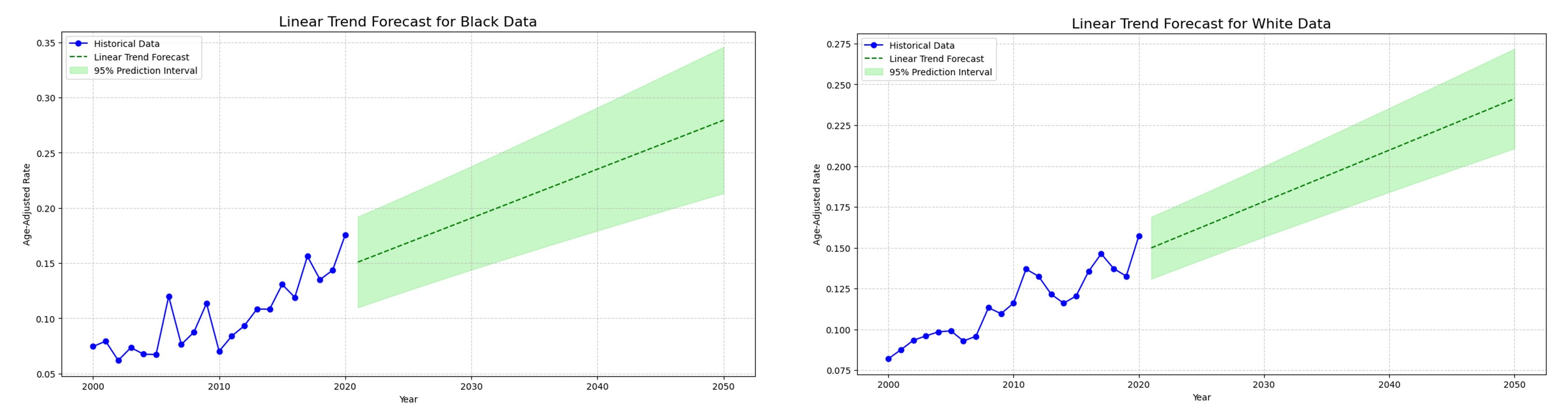

Supplement: Supplementary file 8 — Supplementary Figure 8 (JPG 572 KB) [file 12311_2026_2046_MOESM8_ESM.jpg]

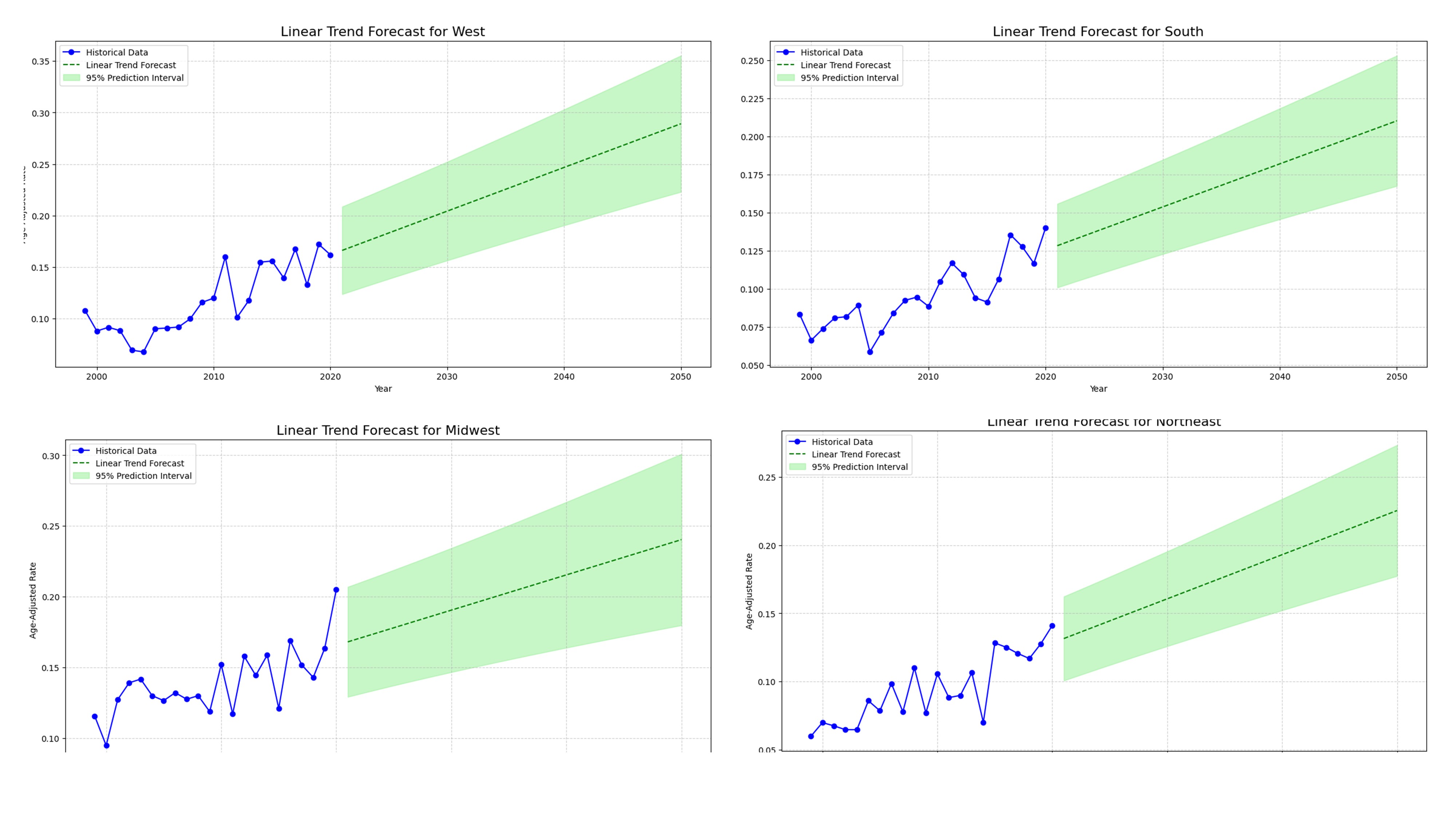

Supplement: Supplementary file 9 — Supplementary Figure 9 (JPG 566 KB) [file 12311_2026_2046_MOESM9_ESM.jpg]

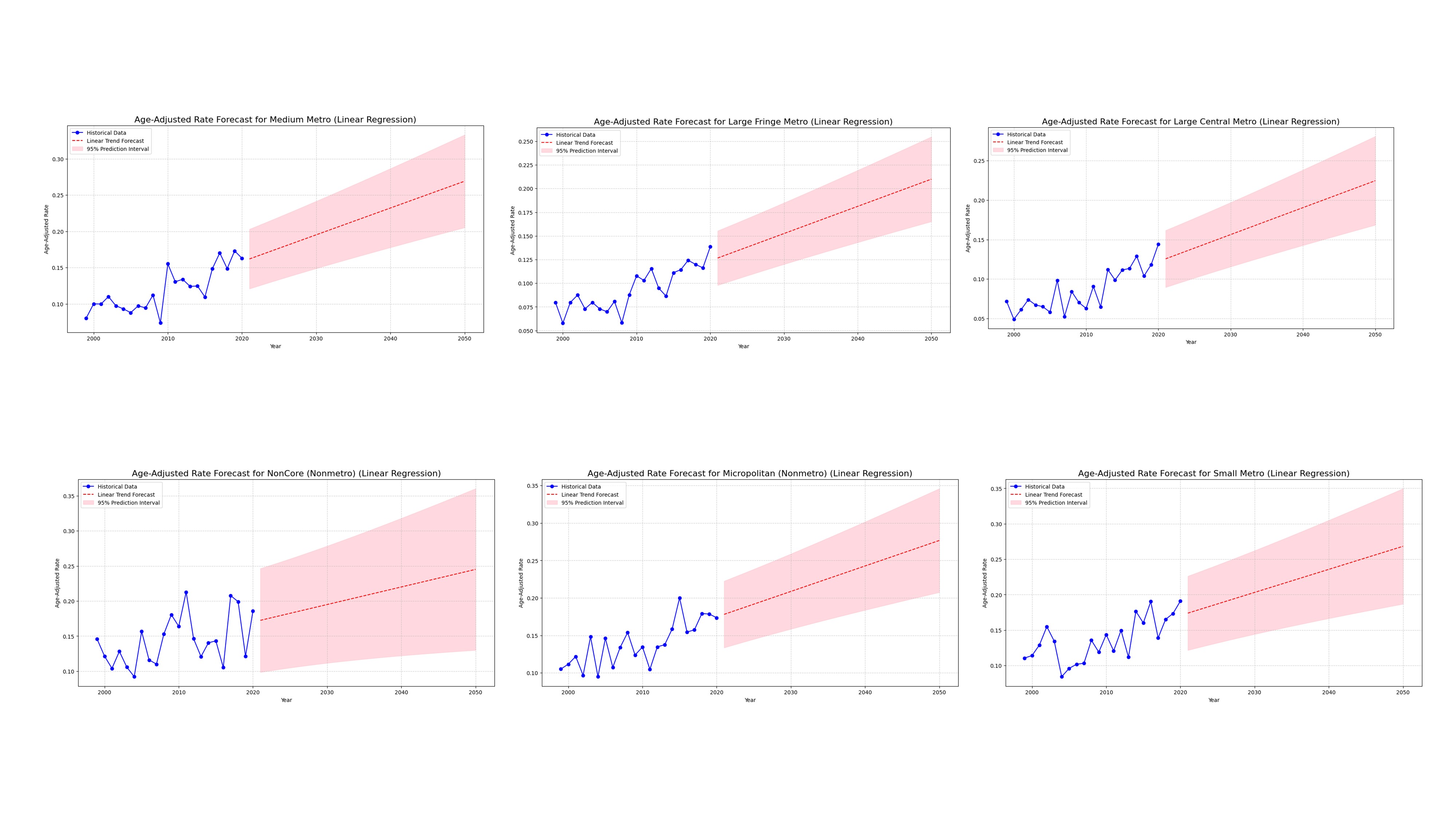

Supplement: Supplementary file 10 — Supplementary Figure 10 (JPG 574 KB) [file 12311_2026_2046_MOESM10_ESM.jpg]
