# Supplementary material for: Trends and Disparities in Mortality from Hereditary Ataxia in United States, 2000–2020: A Retrospective Analysis with Projections to 2050
Source: Cerebellum. 2026 Jun 29;25(4):101. doi: 10.1007/s12311-026-02046-7 (PMC13314688; doi:10.1007/s12311-026-02046-7)
Supplement: Supplementary file 11 — Supplementary File 1 (DOCX 28.5 KB) [file 12311_2026_2046_MOESM11_ESM.docx]

**Supplementary Table 1.** AAMR Change for Hereditary Ataxia by Demographic Subgroup (2000–2020)

| Variable | AAMR  in 2000 | AAMR  in 2020 |
| --- | --- | --- |
| Overall | 0.0805 | 0.157 |
| Sex |  |  |
| Male | 0.0927 | 0.1862 |
| Female | 0.0867 | 0.1325 |
| Census Region |  |  |
| Northeast | 0.07 | 0.1411 |
| Midwest | 0.0949 | 0.205 |
| South | 0.0664 | 0.14 |
| West | 0.0878 | 0.1618 |
| Race |  |  |
| Black/African Americans | 0.0745 | 0.1757 |
| White | 0.082 | 0.1571 |
| Urbanization |  |  |
| Large Central | 0.0490 | 0.1443 |
| Large Fringe | 0.0578 | 0.1385 |
| Medium Metro | 0.1001 | 0.1629 |
| Small Metro | 0.1141 | 0.1910 |
| Micropolitan | 0.1116 | 0.1736 |
| Noncore | 0.1214 | 0.1859 |

**Supplemental Table 2**. Age-Adjusted Mortality Rates Attributed to Hereditary Ataxia in the United States, by Sex (2000–2020)

| Year | Overall AAMR | Male AAMR | Female AAMR |
| --- | --- | --- | --- |
| 2000 | 0.0805 | 0.0927 | 0.0867 |
| 2001 | 0.0859 | 0.1022 | 0.0871 |
| 2002 | 0.0851 | 0.1247 | 0.0789 |
| 2003 | 0.096 | 0.1163 | 0.0996 |
| 2004 | 0.0807 | 0.0994 | 0.0833 |
| 2005 | 0.0925 | 0.1154 | 0.0705 |
| 2006 | 0.0913 | 0.1232 | 0.0781 |
| 2007 | 0.0898 | 0.1018 | 0.0766 |
| 2008 | 0.1051 | 0.1119 | 0.0945 |
| 2009 | 0.1169 | 0.1284 | 0.0876 |
| 2010 | 0.1007 | 0.1537 | 0.0879 |
| 2011 | 0.1189 | 0.1265 | 0.0951 |
| 2012 | 0.1265 | 0.1401 | 0.0840 |
| 2013 | 0.12 | 0.1399 | 0.0895 |
| 2014 | 0.11 | 0.1376 | 0.1138 |
| 2015 | 0.119 | 0.1627 | 0.1083 |
| 2016 | 0.1296 | 0.1477 | 0.1040 |
| 2017 | 0.1463 | 0.1675 | 0.1104 |
| 2018 | 0.1311 | 0.1251 | 0.1185 |
| 2019 | 0.1415 | 0.1820 | 0.1106 |
| 2020 | 0.157 | 0.1862 | 0.1325 |

**Supplemental Table 3.** Age-Adjusted Mortality Rates for Hereditary Ataxia by Race, United States, 2000-2020

| Year | White AAMR | Black AAMR |
| --- | --- | --- |
| 2000 | 0.0820 | 0.0745 |
| 2001 | 0.0875 | 0.0793 |
| 2002 | 0.0933 | 0.0619 |
| 2003 | 0.0960 | 0.0735 |
| 2004 | 0.0985 | 0.0676 |
| 2005 | 0.0991 | 0.0672 |
| 2006 | 0.0929 | 0.1199 |
| 2007 | 0.0958 | 0.0763 |
| 2008 | 0.1133 | 0.0875 |
| 2009 | 0.1096 | 0.1136 |
| 2010 | 0.1161 | 0.0702 |
| 2011 | 0.1369 | 0.0839 |
| 2012 | 0.1325 | 0.0933 |
| 2013 | 0.1216 | 0.1085 |
| 2014 | 0.1160 | 0.1082 |
| 2015 | 0.1205 | 0.1307 |
| 2016 | 0.1356 | 0.1190 |
| 2017 | 0.1463 | 0.1562 |
| 2018 | 0.1372 | 0.1351 |
| 2019 | 0.1326 | 0.1436 |
| 2020 | 0.1571 | 0.1757 |

**Supplemental Table 4.** Age-Adjusted Mortality Rates Attributed to Hereditary Ataxia in the United States, by Census Region (2000–2020)

| Year | Northeast | Midwest | South | West |
| --- | --- | --- | --- | --- |
| 2000 | 0.070 | 0.0949 | 0.0664 | 0.0878 |
| 2001 | 0.0676 | 0.1273 | 0.074 | 0.0914 |
| 2002 | 0.0648 | 0.1392 | 0.081 | 0.0881 |
| 2003 | 0.0648 | 0.1418 | 0.0818 | 0.0692 |
| 2004 | 0.086 | 0.1301 | 0.0894 | 0.0675 |
| 2005 | 0.0786 | 0.1266 | 0.0587 | 0.09 |
| 2006 | 0.0986 | 0.1322 | 0.0715 | 0.0907 |
| 2007 | 0.0779 | 0.1276 | 0.0841 | 0.0917 |
| 2008 | 0.1102 | 0.1301 | 0.0925 | 0.0997 |
| 2009 | 0.0771 | 0.1188 | 0.0946 | 0.1156 |
| 2010 | 0.1056 | 0.1521 | 0.0886 | 0.1197 |
| 2011 | 0.0883 | 0.1172 | 0.1047 | 0.1598 |
| 2012 | 0.0899 | 0.1579 | 0.1169 | 0.1009 |
| 2013 | 0.1065 | 0.1444 | 0.1094 | 0.1173 |
| 2014 | 0.0701 | 0.1588 | 0.0942 | 0.1547 |
| 2015 | 0.1283 | 0.1211 | 0.0914 | 0.1558 |
| 2016 | 0.1251 | 0.1690 | 0.1065 | 0.1393 |
| 2017 | 0.1206 | 0.1518 | 0.1355 | 0.1677 |
| 2018 | 0.1169 | 0.1430 | 0.1278 | 0.1326 |
| 2019 | 0.1277 | 0.1637 | 0.1168 | 0.1720 |
| 2020 | 0.1411 | 0.2050 | 0.1400 | 0.1618 |

**Supplemental Table 5.** Age-Adjusted Mortality Rates Attributed to Hereditary Ataxia in the United States, by Urbanization Level (1999–2020)

| Year | Large Central Metro | Large Fringe Metro | Medium Metro | Small Metro | Micropolitan | Noncore |
| --- | --- | --- | --- | --- | --- | --- |
| 2000 | 0.0490 | 0.0578 | 0.1001 | 0.1141 | 0.1116 | 0.1214 |
| 2001 | 0.0613 | 0.0796 | 0.0999 | 0.1290 | 0.1219 | 0.1039 |
| 2002 | 0.0739 | 0.0875 | 0.1101 | 0.1549 | 0.0967 | 0.1282 |
| 2003 | 0.0672 | 0.0729 | 0.0975 | 0.1343 | 0.1482 | 0.1057 |
| 2004 | 0.0650 | 0.0797 | 0.0932 | 0.0843 | 0.0949 | 0.0921 |
| 2005 | 0.0581 | 0.0729 | 0.0880 | 0.0960 | 0.1462 | 0.1567 |
| 2006 | 0.0983 | 0.0698 | 0.0973 | 0.1018 | 0.1074 | 0.1160 |
| 2007 | 0.0525 | 0.0808 | 0.0946 | 0.1032 | 0.1341 | 0.1100 |
| 2008 | 0.0841 | 0.0582 | 0.1123 | 0.1357 | 0.1539 | 0.1531 |
| 2009 | 0.0702 | 0.0876 | 0.0739 | 0.1189 | 0.1238 | 0.1805 |
| 2010 | 0.0628 | 0.1078 | 0.1552 | 0.1436 | 0.1345 | 0.1639 |
| 2011 | 0.0906 | 0.1029 | 0.1306 | 0.1207 | 0.1047 | 0.2128 |
| 2012 | 0.0648 | 0.1155 | 0.1339 | 0.1496 | 0.1347 | 0.1462 |
| 2013 | 0.1121 | 0.0948 | 0.1242 | 0.1118 | 0.1376 | 0.1208 |
| 2014 | 0.0987 | 0.0864 | 0.1247 | 0.1765 | 0.1586 | 0.1404 |
| 2015 | 0.1115 | 0.1111 | 0.1096 | 0.1601 | 0.2002 | 0.1432 |
| 2016 | 0.1134 | 0.1143 | 0.1485 | 0.1903 | 0.1545 | 0.1053 |
| 2017 | 0.1290 | 0.1242 | 0.1703 | 0.1389 | 0.1574 | 0.2077 |
| 2018 | 0.1038 | 0.1199 | 0.1487 | 0.1652 | 0.1792 | 0.1991 |
| 2019 | 0.1183 | 0.1162 | 0.1731 | 0.1734 | 0.1785 | 0.1212 |
| 2020 | 0.1443 | 0.1385 | 0.1629 | 0.1910 | 0.1736 | 0.1859 |
