# Supplementary material for: Trends and Disparities in Mortality from Hereditary Ataxia in United States, 2000–2020: A Retrospective Analysis with Projections to 2050
Source: Cerebellum. 2026 Jun 29;25(4):101. doi: 10.1007/s12311-026-02046-7 (PMC13314688; doi:10.1007/s12311-026-02046-7)
Supplement: Supplementary file 12 — Supplementary File 2 (DOCX 35.9 KB) [file 12311_2026_2046_MOESM12_ESM.docx]

**Supplementary Table 8. ARIMA Forecast for Overall Hereditary‑Ataxia Mortality (2021 – 2050)**

| Year | Forecasted Rate | Lower 95 % CI | Upper 95 % CI |
| --- | --- | --- | --- |
| 2021 | 0.152192 | 0.133401 | 0.170983 |
| 2022 | 0.155322 | 0.134936 | 0.175708 |
| 2023 | 0.158452 | 0.136587 | 0.180317 |
| 2024 | 0.161582 | 0.138332 | 0.184833 |
| 2025 | 0.164712 | 0.140154 | 0.189270 |
| 2026 | 0.167842 | 0.142043 | 0.193641 |
| 2027 | 0.170972 | 0.143989 | 0.197955 |
| 2028 | 0.174102 | 0.145985 | 0.202219 |
| 2029 | 0.177232 | 0.148025 | 0.206440 |
| 2030 | 0.180362 | 0.150104 | 0.210621 |
| 2031 | 0.183492 | 0.152218 | 0.214766 |
| 2032 | 0.186622 | 0.154364 | 0.218880 |
| 2033 | 0.189752 | 0.156540 | 0.222965 |
| 2034 | 0.192882 | 0.158742 | 0.227023 |
| 2035 | 0.196012 | 0.160968 | 0.231056 |
| 2036 | 0.199142 | 0.163218 | 0.235067 |
| 2037 | 0.202272 | 0.165488 | 0.239056 |
| 2038 | 0.205402 | 0.167778 | 0.243026 |
| 2039 | 0.208532 | 0.170087 | 0.246978 |
| 2040 | 0.211662 | 0.172412 | 0.250912 |
| 2041 | 0.214792 | 0.174754 | 0.254831 |
| 2042 | 0.217922 | 0.177111 | 0.258734 |
| 2043 | 0.221052 | 0.179482 | 0.262622 |
| 2044 | 0.224182 | 0.181867 | 0.266497 |
| 2045 | 0.227312 | 0.184265 | 0.270360 |
| 2046 | 0.230442 | 0.186675 | 0.274210 |
| 2047 | 0.233572 | 0.189097 | 0.278048 |
| 2048 | 0.236702 | 0.191530 | 0.281875 |
| 2049 | 0.239833 | 0.193973 | 0.285692 |
| 2050 | 0.242963 | 0.196427 | 0.289498 |

All rates expressed as age‑adjusted deaths per 100 000 population; forecast generated with best‑fit ARIMA model, 95 % prediction intervals (PI) shown.

**Supplementary Table 9. ARIMA Forecast for Sex Stratified Hereditary Ataxia Mortality (2021 – 2050)**

| Year | Male | Female |
| --- | --- | --- |
| 2021 | 0.172658 | 0.130793 |
| 2022 | 0.176259 | 0.129479 |
| 2023 | 0.179860 | 0.137490 |
| 2024 | 0.183461 | 0.140435 |
| 2025 | 0.187062 | 0.142280 |
| 2026 | 0.190663 | 0.146750 |
| 2027 | 0.194264 | 0.150283 |
| 2028 | 0.197865 | 0.153249 |
| 2029 | 0.201466 | 0.156892 |
| 2030 | 0.205067 | 0.160407 |
| 2031 | 0.208668 | 0.163716 |
| 2032 | 0.212269 | 0.167185 |
| 2033 | 0.215870 | 0.170653 |
| 2034 | 0.219471 | 0.174057 |
| 2035 | 0.223072 | 0.177496 |
| 2036 | 0.226673 | 0.180942 |
| 2037 | 0.230274 | 0.184370 |
| 2038 | 0.233875 | 0.187804 |
| 2039 | 0.237476 | 0.191243 |
| 2040 | 0.241077 | 0.194676 |
| 2041 | 0.244678 | 0.198111 |
| 2042 | 0.248279 | 0.201547 |
| 2043 | 0.251880 | 0.204982 |
| 2044 | 0.255481 | 0.208417 |
| 2045 | 0.259082 | 0.211852 |
| 2046 | 0.262683 | 0.215288 |
| 2047 | 0.266284 | 0.218723 |
| 2048 | 0.269885 | 0.222158 |
| 2049 | 0.273486 | 0.225593 |
| 2050 | 0.277087 | 0.229028 |

Rates expressed as age‑adjusted deaths per 100 000; 95 % prediction intervals (PI) shown.

**Supplementary Table 10. ARIMA Forecast for Hereditary Ataxia Mortality Stratified by Race (2021 – 2050)**

| Year | Black | White |
| --- | --- | --- |
| 2021 | 0.1657 | 0.1687 |
| 2022 | 0.1813 | 0.1592 |
| 2023 | 0.1976 | 0.1587 |
| 2024 | 0.2012 | 0.1633 |
| 2025 | 0.2168 | 0.1667 |
| 2026 | 0.2297 | 0.1703 |
| 2027 | 0.2400 | 0.1739 |
| 2028 | 0.2551 | 0.1775 |
| 2029 | 0.2686 | 0.1811 |
| 2030 | 0.2822 | 0.1847 |
| 2031 | 0.2977 | 0.1883 |
| 2032 | 0.3127 | 0.1919 |
| 2033 | 0.3284 | 0.1955 |
| 2034 | 0.3450 | 0.1991 |
| 2035 | 0.3616 | 0.2027 |
| 2036 | 0.3789 | 0.2063 |
| 2037 | 0.3968 | 0.2099 |
| 2038 | 0.4151 | 0.2135 |
| 2039 | 0.4340 | 0.2171 |
| 2040 | 0.4533 | 0.2207 |
| 2041 | 0.4731 | 0.2243 |
| 2042 | 0.4935 | 0.2279 |
| 2043 | 0.5143 | 0.2315 |
| 2044 | 0.5357 | 0.2351 |
| 2045 | 0.5575 | 0.2387 |
| 2046 | 0.5799 | 0.2423 |
| 2047 | 0.6028 | 0.2459 |
| 2048 | 0.6262 | 0.2494 |
| 2049 | 0.6500 | 0.2530 |
| 2050 | 0.6744 | 0.2566 |

**Supplementary Table 11. ARIMA Forecast for Hereditary Ataxia Mortality Stratified by Census Region (2021 – 2050)**

| Year | Northeast | Midwest | South | West |
| --- | --- | --- | --- | --- |
| 2021 | 0.1332 | 0.1682 | 0.1573 | 0.1692 |
| 2022 | 0.1420 | 0.1707 | 0.1568 | 0.1737 |
| 2023 | 0.1434 | 0.1732 | 0.1448 | 0.1781 |
| 2024 | 0.1480 | 0.1757 | 0.1453 | 0.1826 |
| 2025 | 0.1512 | 0.1782 | 0.1620 | 0.1871 |
| 2026 | 0.1551 | 0.1807 | 0.1736 | 0.1915 |
| 2027 | 0.1586 | 0.1832 | 0.1673 | 0.1960 |
| 2028 | 0.1623 | 0.1857 | 0.1585 | 0.2004 |
| 2029 | 0.1660 | 0.1881 | 0.1659 | 0.2049 |
| 2030 | 0.1696 | 0.1906 | 0.1824 | 0.2093 |
| 2031 | 0.1733 | 0.1931 | 0.1873 | 0.2138 |
| 2032 | 0.1769 | 0.1956 | 0.1783 | 0.2182 |
| 2033 | 0.1805 | 0.1981 | 0.1749 | 0.2227 |
| 2034 | 0.1842 | 0.2006 | 0.1870 | 0.2272 |
| 2035 | 0.1878 | 0.2031 | 0.2004 | 0.2316 |
| 2036 | 0.1915 | 0.2056 | 0.1994 | 0.2361 |
| 2037 | 0.1951 | 0.2081 | 0.1910 | 0.2405 |
| 2038 | 0.1988 | 0.2106 | 0.1934 | 0.2450 |
| 2039 | 0.2024 | 0.2131 | 0.2076 | 0.2494 |
| 2040 | 0.2061 | 0.2156 | 0.2161 | 0.2539 |
| 2041 | 0.2097 | 0.2180 | 0.2111 | 0.2584 |
| 2042 | 0.2134 | 0.2205 | 0.2057 | 0.2628 |
| 2043 | 0.2170 | 0.2230 | 0.2133 | 0.2673 |
| 2044 | 0.2207 | 0.2255 | 0.2267 | 0.2717 |
| 2045 | 0.2243 | 0.2280 | 0.2299 | 0.2762 |
| 2046 | 0.2280 | 0.2305 | 0.2234 | 0.2806 |
| 2047 | 0.2316 | 0.2330 | 0.2225 | 0.2851 |
| 2048 | 0.2353 | 0.2355 | 0.2334 | 0.2895 |
| 2049 | 0.2389 | 0.2380 | 0.2438 | 0.2940 |
| 2050 | 0.2426 | 0.2405 | 0.2427 | 0.2985 |

**Supplementary Table 12. ARIMA Forecast for Hereditary Ataxia Mortality Stratified by Urbanization (2021 – 2050)**

| Year | Large Central Metro | Large Fringe Metro | Medium Metro | Small Metro | Micropolitan | Non‑core |
| --- | --- | --- | --- | --- | --- | --- |
| 2021 | 0.119285 | 0.139048 | 0.178670 | 0.179669 | 0.182279 | 0.173560 |
| 2022 | 0.143860 | 0.145125 | 0.186661 | 0.182826 | 0.182287 | 0.176064 |
| 2023 | 0.139835 | 0.151468 | 0.195007 | 0.185983 | 0.187832 | 0.178568 |
| 2024 | 0.142379 | 0.158077 | 0.203709 | 0.189139 | 0.189842 | 0.181073 |
| 2025 | 0.151883 | 0.164951 | 0.212767 | 0.192296 | 0.194109 | 0.183577 |
| 2026 | 0.150617 | 0.172090 | 0.222181 | 0.195453 | 0.196934 | 0.186082 |
| 2027 | 0.157589 | 0.179496 | 0.231951 | 0.198610 | 0.200680 | 0.188586 |
| 2028 | 0.160940 | 0.187167 | 0.242077 | 0.201766 | 0.203838 | 0.191091 |
| 2029 | 0.164159 | 0.195103 | 0.252559 | 0.204923 | 0.207372 | 0.193595 |
| 2030 | 0.169303 | 0.203306 | 0.263397 | 0.208080 | 0.210666 | 0.196099 |
| 2031 | 0.172426 | 0.211774 | 0.274591 | 0.211237 | 0.214113 | 0.198604 |
| 2032 | 0.176790 | 0.220507 | 0.286141 | 0.214393 | 0.217462 | 0.201108 |
| 2033 | 0.180805 | 0.229507 | 0.298047 | 0.217550 | 0.220873 | 0.203613 |
| 2034 | 0.184588 | 0.238772 | 0.310309 | 0.220707 | 0.224245 | 0.206117 |
| 2035 | 0.188794 | 0.248302 | 0.322927 | 0.223863 | 0.227642 | 0.208622 |
| 2036 | 0.192656 | 0.258099 | 0.335901 | 0.227020 | 0.231023 | 0.211126 |
| 2037 | 0.196683 | 0.268160 | 0.349230 | 0.230177 | 0.234415 | 0.213630 |
| 2038 | 0.200700 | 0.278488 | 0.362916 | 0.233334 | 0.237799 | 0.216135 |
| 2039 | 0.204646 | 0.289081 | 0.376958 | 0.236490 | 0.241188 | 0.218639 |
| 2040 | 0.208674 | 0.299940 | 0.391356 | 0.239647 | 0.244574 | 0.221144 |
| 2041 | 0.212649 | 0.311065 | 0.406109 | 0.242804 | 0.247962 | 0.223648 |
| 2042 | 0.216641 | 0.322455 | 0.421219 | 0.245961 | 0.251349 | 0.226153 |
| 2043 | 0.220640 | 0.334111 | 0.436685 | 0.249117 | 0.254737 | 0.228657 |
| 2044 | 0.224623 | 0.346032 | 0.452506 | 0.252274 | 0.258124 | 0.231161 |
| 2045 | 0.228620 | 0.358219 | 0.468684 | 0.255431 | 0.261511 | 0.233666 |
| 2046 | 0.232610 | 0.370672 | 0.485217 | 0.258587 | 0.264898 | 0.236170 |
| 2047 | 0.236601 | 0.383391 | 0.502107 | 0.261744 | 0.268286 | 0.238675 |
| 2048 | 0.240594 | 0.396375 | 0.519352 | 0.264901 | 0.271673 | 0.241179 |
| 2049 | 0.244584 | 0.409624 | 0.536954 | 0.268058 | 0.275060 | 0.243684 |
| 2050 | 0.248576 | 0.423140 | 0.554911 | 0.271214 | 0.278447 | 0.246188 |

**Supplementary Table 13. ARIMA Models for Forecast for Hereditary Ataxia Mortality of Different Strata (2021 – 2050)**

| Stratum | ARIMA Model |
| --- | --- |
| Overall | (0, 1, 1) |
| Gender | |
| Male | (0, 1, 1) |
| Female | (2, 2, 1) |
| Race | |
| Black | (2, 2, 1) |
| White | (1, 2, 4) |
| Census Region | |
| Northeast | (1, 2, 2) |
| Midwest | (0, 1, 1) |
| South | (2, 1, 2) |
| West | (0, 2, 2) |
| Urbanization | |
| Large Central Metro | (2, 2, 1) |
| Large Fringe Metro | (0, 2, 2) |
| Medium Metro | (0, 2, 2) |
| Small Metro | (0, 1, 1) |
| Micropolitan | (1, 2, 1) |
| Non‑core | (0, 2, 2) |
