# Supplementary material for: Trends and Disparities in Mortality from Hereditary Ataxia in United States, 2000–2020: A Retrospective Analysis with Projections to 2050
Source: Cerebellum. 2026 Jun 29;25(4):101. doi: 10.1007/s12311-026-02046-7 (PMC13314688; doi:10.1007/s12311-026-02046-7)
Supplement: Supplementary file 13 — Supplementary File 3 (DOCX 15.5 KB) [file 12311_2026_2046_MOESM13_ESM.docx]

Supplementary Table 12. Newly available observed CDC WONDER mortality rates for 2021 to 2024.

| Notes | Year | Deaths | Population | Age Adjusted Rate | Age Adjusted Rate Lower 95% Confidence Interval | Age Adjusted Rate Upper 95% Confidence Interval | Age Adjusted Rate Standard Error | % of Total Deaths |
| --- | --- | --- | --- | --- | --- | --- | --- | --- |
|  | 2021 | 609 | 331893745 | 0.1432 | 0.1308 | 0.1566 | 0.0065 | 14.23% |
|  | 2022 | 635 | 333287557 | 0.1581 | 0.1455 | 0.1715 | 0.0065 | 14.84% |
|  | 2023 | 650 | 334914895 | 0.1601 | 0.1477 | 0.1733 | 0.0064 | 15.19% |
|  | 2024 | 707 | 340110988 | 0.1749 | 0.1623 | 0.1885 | 0.0066 | 16.52% |
| Total |  | 4280 | 2325098265 | 0.1504 | 0.1457 | 0.1552 | 0.0024 | 100.00% |
