# Supplementary material for: Trends and Disparities in Mortality from Hereditary Ataxia in United States, 2000–2020: A Retrospective Analysis with Projections to 2050
Source: Cerebellum. 2026 Jun 29;25(4):101. doi: 10.1007/s12311-026-02046-7 (PMC13314688; doi:10.1007/s12311-026-02046-7)
Supplement: Supplementary file 14 — Supplementary File 4 (DOCX 16.1 KB) [file 12311_2026_2046_MOESM14_ESM.docx]

**Exact CDC WONDER query parameters.**

- **Database:** Multiple Cause of Death, 1999 to 2020, deaths occurring from 2000 to 2020
- **Group Results By:** Year
- **Measures selected:** Deaths, Population, Age-Adjusted Rate, 95% Confidence Interval, Standard Error, Percent of Total Deaths
- **Rates calculated per:** 100,000
- **Standard population:** 2000 U.S. Standard Population
- **Location:** All U.S. states, effectively national data
- **Urbanization scheme visible:** 2013 Urbanization
- **Demographics:** All ages, All sexes, All races, All Hispanic origins, with subgroup queries then generated separately
- **Underlying cause of death:** All causes of death
- **Multiple cause of death selection:** G11 (Hereditary ataxia)
- **Export options:** XLS, show totals, show zero values, show suppressed values, precision 4 decimal places
